# Multi‐Objective Bayesian Optimization for Laminate‐Inspired Mechanically Reinforced Piezoelectric Self‐Powered Sensing Yarns

**DOI:** 10.1002/advs.202402440

**Published:** 2024-06-27

**Authors:** Ziyue Yang, Kundo Park, Jisoo Nam, Jaewon Cho, Yong Jun Choi, Yong‐Il Kim, Hyeonsoo Kim, Seunghwa Ryu, Miso Kim

**Affiliations:** ^1^ Department of Advanced Materials Science and Engineering Sungkyunkwan University (SKKU) Suwon 16419 Republic of Korea; ^2^ Department of Mechanical Engineering Korea Advanced Institute of Science and Technology (KAIST) Daejeon 34141 Republic of Korea; ^3^ Department of Mechanical Engineering University of California Berkeley CA 94720 USA; ^4^ Korea Research Institute of Standards and Science Daejeon 34113 Republic of Korea; ^5^ SKKU Institute of Energy Science and Technology (SIEST) Sungkyunkwan University (SKKU) Suwon 16419 South Korea

**Keywords:** electrospinning, machine learning, multi‐objective Bayesian optimization, piezoelectric yarns, self‐powered sensing

## Abstract

Piezoelectric fiber yarns produced by electrospinning offer a versatile platform for intelligent devices, demonstrating mechanical durability and the ability to convert mechanical strain into electric signals. While conventional methods involve twisting a single poly(vinylidene fluoride‐co‐trifluoroethylene)(P(VDF‐TrFE)) fiber mat to create yarns, by limiting control over the mechanical properties, an approach inspired by composite laminate design principles is proposed for strengthening. By stacking multiple electrospun mats in various sequences and twisting them into yarns, the mechanical properties of P(VDF‐TrFE) yarn structures are efficiently optimized. By leveraging a multi‐objective Bayesian optimization‐based machine learning algorithm without imposing specific stacking restrictions, an optimal stacking sequence is determined that simultaneously enhances the ultimate tensile strength (UTS) and failure strain by considering the orientation angles of each aligned fiber mat as discrete design variables. The conditions on the Pareto front that achieve a balanced improvement in both the UTS and failure strain are identified. Additionally, applying corona poling induces extra dipole polarization in the yarn state, successfully fabricating mechanically robust and high‐performance piezoelectric P(VDF‐TrFE) yarns. Ultimately, the mechanically strengthened piezoelectric yarns demonstrate superior capabilities in self‐powered sensing applications, particularly in challenging environments and sports scenarios, substantiating their potential for real‐time signal detection.

## Graphical abstract

Inspired by composite laminates, electrospun fiber mats are strategically stacked to create highly strengthened P(VDF‐TrFE) yarns, preserving their high piezoelectric performance. A multi‐objective Bayesian optimization‐based machine learning algorithm is developed to optimize the stacking sequence, ultimately yielding mechanically robust piezoelectric polymer yarn with simultaneously improved ultimate tensile strength and failure strain for real‐time self‐powered sensing applications in diverse environmental conditions.

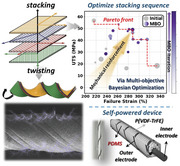

## 1 Introduction

Piezoelectric polymer fibers possess remarkable attributes such as high shape conformability, facile processability, and mechanical flexibility, making them well‐suited for versatile, wearable, and biomedical self‐powered sensing applications.^[^ ^1^ ^]^ These fibers exhibit desirable mechanical properties, including stretchability and biocompatibility. Notably, the synthesis of polyvinylidene fluoride (PVDF) and poly(vinylidene fluoride‐trifluoroethylene) (P(VDF‐TrFE)) fibers through a single‐step electrospinning process capitalizes on the in situ electrical poling and mechanical stretching effects, coupled with the geometric confinement effect inherent in the fiber, facilitating the formation of a highly crystalline *β* responsible for piezoelectricity. Despite these advantages, piezoelectric polymer fibers exhibit poorer piezoelectric properties than their inorganic counterparts, prompting extensive research to enhance their performance. Material‐based strategies involve incorporating inorganic or conductive nanofillers, such as barium titanate (BaTiO_3_)^[^ ^2^ ^]^ or zinc oxide (ZnO)^[^ ^3^ ^]^ nanoparticles, silver nanowires,^[^ ^4^ ^]^ graphene oxide,^[^ ^5^ ^]^ multi‐walled carbon nanotubes,^[^ ^6^ ^]^ MXenes,^[^ ^7^ ^]^ or ionic surfactants^[^ ^8^ ^]^ into the polymer fibers. This approach stabilizes the all‐trans conformation locally via electrostatic interactions, promoting *β*‐phase formation.^[^ ^9^ ^]^ Other studies have explored the selection of piezoelectrically optimal solvents based on their compatibility with polymer matrices.^[^ ^10^ ^]^ Postprocessing techniques, including direct current (DC) or corona poling and thermal annealing, have also been investigated to enhance the crystallization of *β*‐phase.^[^ ^11^ ^]^ Structural modifications, such as designing a collector for electrospinning or an electrode configuration, have been proven to result in a higher degree of fiber alignment and overall enhanced piezoelectricity.^[^ ^12^ ^]^

Further structuring of electrospun fiber mats into yarns, coils, or textiles significantly enhances their mechanical robustness and broadens their potential for large‐scale utilization. The two primary approaches for creating PVDF or P(VDF‐TrFE) yarns involve mechanically twisting the electrospun mat or coating the core‐sheath material with a piezoelectric polymer via electrospinning, followed by subsequent twisting or weaving to create yarn‐type or fabric piezoelectric devices.^[^ ^13^ ^]^ These mechanically reinforced piezoelectric yarn devices demonstrated versatility and are employed in applications such as intelligent human motion sensing for machine learning classification^[^ ^14^ ^]^ and acoustic‐to‐electrical signal transduction in acoustic fabrics for fiber computing applications.^[^ ^15^ ^]^ While some studies have explored the properties and applicability of electrospun fiber‐derived piezoelectric polymer yarns, significant opportunities exist to enhance their performance compared to the extensively studied electrospun fiber mats. Notably, the piezoelectric performance of the aligned fiber mats surpasses that of random mats, both parallel and perpendicular to the alignment direction, highlighting the superior characteristics of using aligned mats to create yarns.^[^ ^16^ ^]^ However, the mechanical properties exhibit anisotropic behavior based on the angle between the fiber alignment and tensile directions.^[^ ^17^ ^]^ Aligned fiber mats that were twisted into yarns inevitably deviated from the axis direction during the twisting process, resulting in a less optimal mix of anisotropic mechanical properties inherited from the original fiber mat. Consequently, the high mechanical strength of the electrospun fiber mats does not necessarily translate into superior mechanical performance of the electrospun fiber‐based piezoelectric yarns. This underscores the need for further research to identify the optimal mat conditions from a yarn perspective.

The strengthening mechanisms inherent in composite laminates can offer a solution for tailoring the mechanical properties of electrospun fiber‐derived yarns of the piezoelectric polymer P(VDF‐TrFE). A composite laminate is structured with multiple layers or plies, each composed of unidirectional fibers and a matrix. The factors that influence the mechanical properties of such composite laminates include the material properties of the plies, number of plies, ply thickness, fiber orientation, and stacking sequence of the plies.^[^ ^18^ ^]^ Our attention is particularly focused on fiber orientation and stacking sequence. By controlling the orientation of the aligned fibers, high mechanical strength can be achieved in specific directions. The stacking sequence categorizes the composite laminates into various types, such as angle and cross‐ply laminates, which can be symmetric, antisymmetric, or balanced.^[^ ^19^ ^]^ The arrangement of unidirectional fiber layers in the stacking sequence significantly affects the interlaminar stress distribution, leading to varied modes of failure and allowing multifaceted control over the targeted mechanics.^[^ ^20^ ^]^ Furthermore, in multilayer fabrics, angular stacking in quasi‐isotropic laminates enables the primary fibers of one layer to influence the secondary fibers of the next layer. This arrangement provides additional regions for energy dissipation and absorption compared with unidirectionally aligned fabrics.^[^ ^20^, ^21^ ^]^ The optimization of the stacking sequences in composite laminates forms the theoretical basis for our research scheme. Conventionally, electrospinning‐derived piezoelectric yarns involve twisting a single layer of a unidirectional fiber mat, limiting the ability to tailor the mechanical properties during the yarn‐making process once the fiber alignment direction is determined. By contrast, our approach involves constructing a mat comprising a yarn with multiple layers, aligning each layer based on the direction of the aligned fiber mat, thereby creating the yarn and bearing the idea of tailoring the desired mechanical properties (i.e., ultimate tensile strength (UTS) and failure strain).

In this study, we derived inspiration from composite laminate structures and leveraged the diverse stacking sequences achievable through the controlled fiber orientations of electrospun mats. When strategically layered and twisted, these mats formed yarns. By introducing a multi‐objective Bayesian optimization (MBO)‐based machine learning approach, we aimed to optimize the stacking sequence. Although previous studies have explored stacking sequence optimization for composite laminates using genetic algorithms,^[^ ^22^ ^]^ there is a notable absence of such optimization for piezoelectric yarns. Our unique contribution involves the application of a data‐driven optimization framework using machine learning. Specifically, we optimized the objective functions, defined by the UTS and failure strain, for discrete design variables, namely fiber orientation angles. Moreover, in this study, following the fabrication of the yarns, corona poling was employed to induce polarization alignment at the material level, thereby enhancing the piezoelectric properties. Ultimately, mechanically robust yarns exhibiting high piezoelectric performance were obtained. The efficacy of the high‐performance yarn device was validated under harsh environments, demonstrating its proficiency in self‐powered wind monitoring for smart camping tents and touch‐ and pressure‐based self‐powered sensing for smart volleyball nets, highlighting the versatility of the proposed applications.

## 2 Results and Discussion

### 2.1 Laminate‐Inspired Multistacked Piezoelectric Yarns

**Figure 1:**
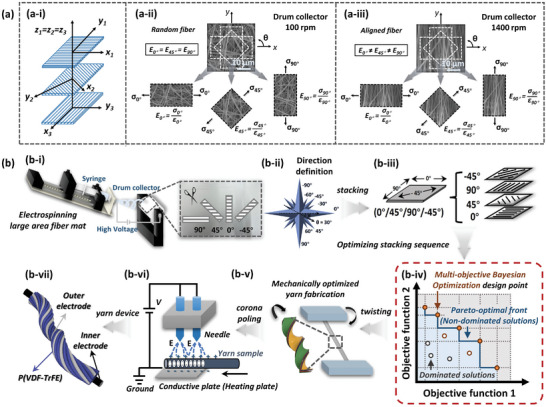
Overall concept of the laminate‐inspired multistacked piezoelectric yarns. a‐i) A configuration of 3‐layer composite laminate for mechanical strengthening. Schematic diagram illustrating the mechanical properties of a‐ii) random electrospun fibers and a‐iii) aligned electrospun fibers under the various tensile directions. (Inset: Scanning electron microscope (SEM) images of random and aligned fiber mats, magnification: × 2500.) b) Schematic diagram of the yarn production and optimization process with the dual improvement of mechanical and piezoelectric properties: b‐i) electrospinning for large‐area fiber mat production, b‐ii) illustration of stacking angles depending on cutting, b‐iii) multilayer stacking operation, b‐iv) MBO‐based machine learning optimization process for stacking sequences, b‐v) schematic representation of the twisting operation, b‐vi) schematic diagram of corona poling process for yarns, and b‐vii) illustration of yarn device.

**Figure** **1**a illustrates the comprehensive concept of stacked yarns inspired by laminates. Figure 1a‐i presents examples of a three‐layer stacked cross‐ply laminate structure. As shown in the figure, the optimal loading direction for each material property varied, with the x‐axis representing the high‐strength direction and the y‐axis indicating the optimal ductility direction. Thus, this strategic cross‐ply arrangement achieved the desired outcome of uniformly demonstrating outstanding mechanical performance in multiple directions. In Figure 1a‐ii,iii, scanning electron microscopy (SEM) images show two types of electrospun P(VDF‐TrFE) fibers: randomly distributed fibers produced using a drum collector at 100 rpm and aligned distributed fibers produced at a much higher rotation speed of 1400 rpm. It is worth noting that according to our previous study,^[^ ^12^ ^]^ the enhanced piezoelectric performance correlates positively with the higher degree of fiber alignment with increasing rotation speeds of the drum collector up to 2000 rpm. However, at above 1500 rpm, too high air flow often induces unstable fiber structures, which is unsuitable for device fabrication. Furthermore, owing to the induced airflow proximate to the surface of the rotating collector, there is also a slight increment in the *β*‐phase content up to a defined threshold, culminating in saturation (above 1000 rpm). Therefore, here we chose the optimal rotation speed of 1400 rpm that can show a sufficient degree of fiber alignment with enhanced *β*‐phase content. In the case of random fibers, when stress was applied in different directions (σ_i°_) on the fiber, the elastic modulus (E_i°_) remained the same in each direction. Consequently, from a mechanical property's perspective, it can be considered as an “isotropic material” (Figure S1, Supporting Information). By contrast, aligned fibers exhibited varying E_i°_ values for each direction, classifying them as an “anisotropic material,” aligning with the requirements for the base material in Figure 1a‐i. These two types of fiber alignments were the basis mat configurations for further yarn production in this study. Figure 1b shows the optimization process for creating the ultimate high‐performance yarns. In the electrospinning stage, Figure 1b‐i, we meticulously controlled various processing parameters, including the environmental relative humidity, solvent type, collector material, rotation speed, concentration of the electrospinning solution, and lateral movement distance of the syringe pump, to ultimately yield large‐area aligned fiber mats exhibiting enhanced piezoelectric properties (Figures S2–S6, Supporting Information). The diameter of the obtained fibers was ≈500 nm (Figure S7, Supporting Information). Subsequently, fiber mats with different orientations were obtained by cutting large‐area fiber mats, with the specific direction definition outlined in Figure 1b‐ii. After confirming that the multilayer stacking operation did not compromise the piezoelectric and mechanical properties of both the mats and yarns compared to the single‐layer‐derived cases (Figures S8 and S9, Supporting Information), we chose four layers of fiber mats for stacking, as depicted in Figure 1b‐iii. With eight discrete angle choices {0°, 30°, −30°, 45°, −45°, 60°, −60°, 90°} available for each layer, a four‐layer stacked fiber mat offers 8^4^ (= 4096) possible combinations. Regarding the angles in each stacking layer, we chose a non‐continuous design space with eight clearly discrete levels. This decision was made because the experimental samples were manually prepared by the researcher, inevitably introducing random errors due to the difficulty of achieving highly precise angles. To minimize the relative impact of these errors in our research, we opted for eight distinct angle levels. Achieving optimal stacking among these numerous possibilities is challenging when using experimental methods alone. Therefore, we adopted an MBO design approach to optimize the stacking sequence. We delineated the Pareto‐optimal front through iterative experimental measurements of the mechanical performance of the MBO‐predicted multistacking designs (Figure 1b‐iv). This approach enabled the selection of an optimal design tailored for specific application scenarios. Here, it should be noted that in our study, the selection of four layers and eight angles was a result of careful consideration encompassing both the laminate stacking mechanism and the MBO optimization algorithm. First, in consideration of the laminate stacking mechanism, adhering to the principles of composite laminate design, we concluded that a minimum of four stacking layers would be essential.^[^ ^23^ ^]^ Second, in relation to the MBO optimization algorithm, our approach integrates a surrogate model within the MBO framework. The volume of data required to uphold a consistent data coverage density, which significantly impacts the predictive model's performance, increases exponentially with the addition of each layer, doubling eight times. Consequently, our selection effectively minimizes the computational resources demanded during the MBO process while simultaneously facilitating ample avenues for the realization of our objectives. After identifying the optimized stacking sequence of the four stacked layers, we fashioned the yarn structure using twisting operations to achieve additional mechanical strengthening (Figure 1b‐v). Subsequently, by directly subjecting the yarn to corona poling, we ensured consistent polarization alignment of dipoles and further enhanced the piezoelectric properties (Figure 1b‐vi). Ultimately, the yarn with simultaneously improved mechanical and piezoelectric properties is processed into a device for specific applications (Figure 1b‐vii).

### 2.2 Piezoelectric and Mechanical Characterization of Fiber Mats

**Figure 2:**
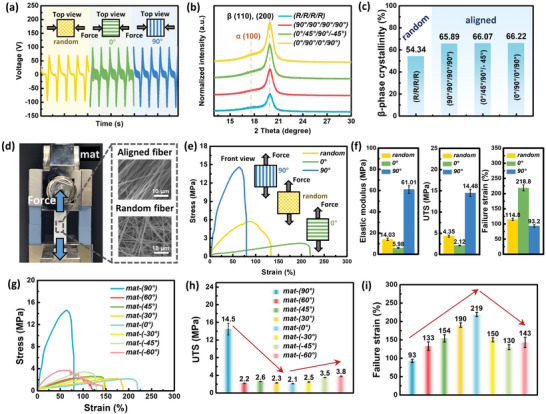
Piezoelectric and mechanical characterization of electrospun fiber mats. a) Voltage output of random fiber mats and aligned fiber mats in different directions under mechanical bending. b) XRD patterns of random‐one layer (R/R/R/R), aligned‐one layer (90°/90°/90°/90°), stacking at different angles‐four‐layer (0°/45°/90°/−45°), and (0°/90°/0°/90°) fiber mats. c) *β*‐phase crystallinity calculated from XRD measurement. d) Digital photos of the tensile test setup and SEM images of aligned and random fiber mats (magnification: × 2500). e) Stress–strain curves of random fiber mats and aligned fiber mats in two different directions, and f) resulting bar graphs of elastic modulus, UTS, and failure strain achieved from (e). g) Stress–strain curves, h) UTS, and i) failure strain of fiber mats at eight different angles.

**Figure** **2** shows the characterization results of the piezoelectric and mechanical properties of the fibers at the electrospun mat level. Our analysis not only involved a comparison between the piezoelectric and mechanical performance of random and aligned fibers but also delved into the specific impact of variations in fiber orientation within aligned fibers on performance. First, the piezoelectric properties of the fiber mats were characterized through mechanical bending tests on mats with two different degrees of alignment, as shown in Figure 2a. A comparison of the results of the random and aligned mats indicated a noticeable increase in the piezoelectric output as the degree of alignment of the fibers increased. Furthermore, bending tests were conducted on the aligned fiber mats in two different directions perpendicular to each other, revealing that the bending direction had little effect on the piezoelectric performance of the mats. All these results were consistent with those of our previous study.^[^ ^12^ ^]^ Subsequently, we progressed from single‐angle fiber mats to stacked multi‐angle fiber mats by stacking random and aligned fibers. The piezoelectric performance of the mats was characterized using X‐ray diffraction (XRD) and Fourier‐transform infrared spectroscopy (FTIR). Given the inherent limitation in the theoretical penetration depth of X‐rays, we integrated insights from our prior work on the penetration characteristics of theoretical X‐rays with the specific attributes of P(VDF‐TrFE) under consideration,^[^ ^24^ ^]^ calculated the theoretical X‐ray penetration depth of P(VDF‐TrFE) (75:25), as depicted in Figure S10 (Supporting Information). Our findings revealed a penetration depth exceeding 100 µm. Therefore, here, we controlled the thickness of each XRD sample to be ≈80 µm and finally normalized the intensity according to the specific sample thickness. Consequently, we ensure that our fiber mats can be adequately penetrated by X‐rays in theory, and our analysis method for calculating the *β* phase is considered sufficient and appropriate. The XRD patterns in Figure 2b confirm the predominant formation of *β* phases in all stacked mats, with characteristic peaks at 2θ = 19.8°−19.9° corresponding to (110) and (200) reflections of the polar *β* phase.^[^ ^10^, ^25^ ^]^ From the XRD patterns, the *β*‐phase crystallinity (%) can be quantitatively defined as the ratio of the amount of crystalline *β*‐phase to the total amount of all existing phases (both crystalline and amorphous phases), as illustrated in Figure S11 (Supporting Information). The *β*‐phase crystallinity (%) from the XRD spectra in Figure 2c and the *β*‐phase content result from the FTIR analysis shown in Figure S12 (Supporting Information), both quantitatively calculated, indicate that the piezoelectric performance of aligned fibers remained consistently higher at a similar level compared to the random mats, irrespective of the stacking angle.

Having verified that the stacking operation preserved the high piezoelectric performance of the aligned fibers, we conducted tensile tests on the single‐angle fiber mat samples to characterize their mechanical performance using the experimental setup depicted in Figure 2d. The tensile test results presented in Figure 2e,f reveal that the aligned fibers display anisotropic mechanical performance depending on the angle between the applied strain and fiber alignment. Specifically, the elastic modulus and UTS of the 90° fiber mat increased significantly compared with those of the random fiber mat, but the failure strain decreased. By contrast, the 0° fiber mat exhibited a substantial increase in failure strain, whereas the elastic modulus and UTS decreased markedly. To further explore the impact of fiber orientation on mechanical performance, tensile tests were also conducted on fiber mats at six other angles (±60°, ±45°, ±30°). In Figure 2g–i, both the UTS and failure strain exhibit a certain trend; the UTS decreased as the absolute value of the fiber orientation angle decreased, whereas the failure strain increased accordingly.

### 2.3 Fabrication and Characterizations of Piezoelectric Yarns

**Figure 3:**
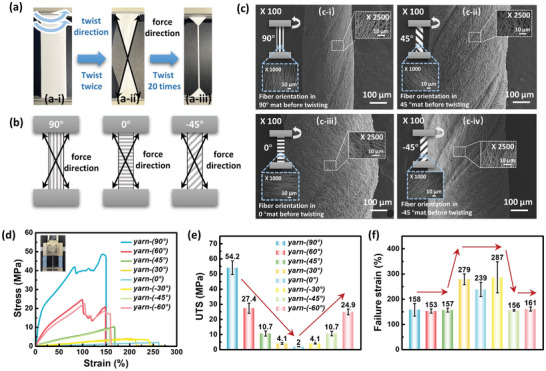
Characterizations of P(VDF‐TrFE) yarns. a) Digital photos of the yarns at different stages: a‐i) initial fiber mat, a‐ii) fiber mat after twisting twice, and a‐iii) fiber mat after twisting 20 times (yarn). b) Force direction in the fiber mat during the yarn‐making process. c) SEM images of the fabricated yarns derived from different fiber orientation angles: c‐i) 90°, c‐ii) 45°, c‐iii) 0°, c‐iv) −45°. Magnification: × 100, Inset top right: Magnification: × 2500, fiber orientation within the yarn. Inset bottom left: Magnification: × 1000, fiber orientation in the fiber mat (before yarn manufacturing). d) Stress–strain curves, e) UTS, and f) failure strain of yarns derived from various angles.

To achieve further mechanical strengthening and enhance the interlayer load transfer after stacking, we executed a twisting operation to produce a yarn structure derived from a single‐layer electrospun fiber mat (**Figure** **3**). The specific twisting process is shown in Figure 3a, where the blue arrows denote the direction of jig rotation. After two rotation cycles, the stress distribution in the fiber mat during the twisting process was observed in a digital photograph (Figure 3a‐ii). After 20 cycles, a yarn sample with a uniform diameter was obtained (Figure 3a‐iii). In line with our prior findings,^[^ ^14^ ^]^ we opted for a rotation speed of 50 rpm for yarn twisting to achieve the highest piezoelectric performance compared to other rotation speeds. The stress distribution during the twisting process (Figure 3b) significantly influenced the final surface morphology of the fiber yarns derived from different orientations, which are showcased in the SEM images of yarns produced from 90°, 45°, 0°, and −45° fiber mats in Figure 3c and those from other angles (±60°, ±30°) in Figure S13 (Supporting Information). The SEM images within the blue dashed insets in Figure 3c show the fiber orientations before the twisting process. We observe that fiber mats at 90°, ±60°, and ±45° maintained their alignment after twisting, while the alignment of 0° and ±30° fibers was disrupted. This disruption is attributed to uneven forces generated during the twisting process owing to our twisting operation following the “dual‐Archimedean” yarn fabrication method.^[^ ^26^ ^]^ This method exerted the maximum force diagonally inside the fiber mat, as illustrated in Figure 3a‐ii,b. Twisting forces did not alter the fiber's alignment when the fiber orientation was perpendicular to the twisting direction (almost parallel to the direction of force, as in the cases of 90°, ±60°, and ±45°). Conversely, when the fiber orientation was parallel to the twisting direction (almost perpendicular to the direction of force, as in the cases of 0° and ±30°), the fibers in the middle part were not stretched, resulting in irregular shapes in SEM images of yarns at 0° and ±30°. Furthermore, the diameter of the yarn was notably influenced by differences in force distribution. The diameters of the yarns with different orientation angles are plotted in Figure S14 (Supporting Information) revealing an increase in the yarn diameter as the absolute value of the fiber orientation angle decreased.

Tensile stress–strain (S‐S) curves are shown in Figure 3d. Note that although each orientation yarn differed in diameter, the S‐S curves were scale‐normalized and thus did not affect the trend. Overall, irrespective of the orientation, the P(VDF‐TrFE) yarns exhibited improved mechanical properties, as shown in Figure 3d,e, compared to the fiber mats (Figure 2g–i). Notably, the difference in the UTS with varying orientation angles became more pronounced in the yarns: 90° > ±60° > ±45° > ±30° > 0° (Figure 3e). The more pronounced difference in the UTS is attributed to the twisting operation. This operation serves to tighten the binding between the fibers, enhance their connection, and facilitate the conduction of loads. Regarding the failure strain, the test results can be divided into two main regions. Following the twisting process, the 90°, ±60°, and ±45° yarns, characterized by minimal disruption to the alignment degree, predominantly manifested a failure strain of ≈160%. By contrast, the 0° and ±30° yarns, which displayed a bent fiber morphology after twisting (see Figure 3c‐iii), showcased superior ductility, with a concentrated failure strain reaching ≈250% (Figure 3f). This trend could be attributed to the fact that for single‐angle yarns, the failure strain primarily depends on the disparity between the direction of the force and the fiber orientation. The failure strain decreases as the fiber orientation approaches a parallel alignment with the direction of the force. Conversely, a greater perpendicular alignment between the fiber orientation and the direction of the force resulted in a larger failure strain and enhanced ductility. This finding reveals a tradeoff relationship between the UTS and failure strain of the yarn sample, which means that pursuing balanced mechanical performance becomes particularly meaningful in the realm of yarns. Upon further examination of the S‐S curves illustrated in Figure 3d, notable observations emerge. Specifically, certain yarns at particular angles exhibit a wavering and unstable pattern in their S‐S curves, for example, the 90° yarn strain range of ≈75–125%. This phenomenon arises due to the alignment of the fiber direction with the tensile direction, where a portion of the fibers fracture prematurely under tension, consequently inducing a sudden reduction in the stress required for elongation. This sudden decline aligns with the abrupt drop observed in the S‐S curve at 90° yarn strain of 75% and ±60° yarn strain of 100%. Conversely, when the fiber orientation is perpendicular to the tensile direction, the applied force primarily functions to separate the fibers, thereby absenting a sudden force drop. Consequently, the stress reduction induced by fiber fracture leads to the observed waviness in the S‐S curves, which is not evident in yarns oriented perpendicular to the direction of tension.

### 2.4 Machine Learning‐Based Optimization of Mechanical Performance for Multilayer Stacked Yarns

**Figure 4:**
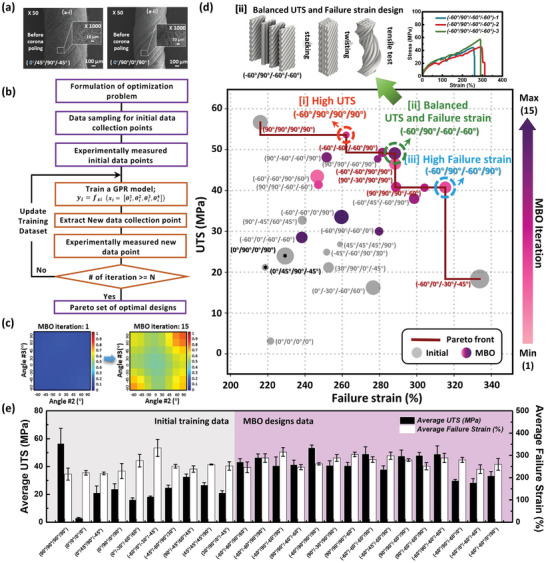
Machine learning‐based optimization of mechanical performance for multilayer stacked yarns. a) SEM images of two representative multistacked yarns: a‐i) (0°/45°/90°/−45°) and a‐ii) (0°/90°/0°/90°) (Magnification: × 50, Inset Magnification: × 1000.) b) Overall workflow chart of the MBO framework. c) 2D representation of the GPR model for ultimate strength at MBO iterations 1 and 15. d) Point distribution map of UTS versus failure strain where initial data and MBO design data are all plotted; Pareto‐optimal front is indicated with a red line. e) Histogram of UTS and failure strain for all data.

Based on the tradeoff relationship between the UTS and failure strain of single‐layer‐derived yarns with different orientations, we attempted to balance these two mechanical properties of piezoelectric yarns through multilayer stacking (**Figure** **4**). Initially, we explored two common stacking sequences used in composite laminate structures: stacking at 45‐degree intervals (0°/45°/90°/−45°) and stacking at 90‐degree intervals (0°/90°/0°/90°).^[^ ^27^ ^]^ Examination of the SEM characterization of these two stacked yarns (Figure 4a) showed that the morphology of the multi‐angle stacked yarns was primarily determined by the outermost layer of fibers, that is, the orientation of the first layer in the stacking sequence. For instance, as illustrated in Figure 4a, the morphology of stacked yarns with (0°/45°/90°/−45°) and (0°/90°/0°/90°) closely resembles that of a 0° single‐angle yarn. Additionally, we compared the SEM images of the failed parts after the tensile tests for the stacked and non‐stacked yarns. The twisting operation effectively integrated the four layers of fibers, eliminating the occurrence of layer‐by‐layer fractures (Figure S15, Supporting Information). While prior research has often focused on specific stacking arrangements for composite laminate structures (such as symmetrical stacking, cross‐stacking, and quasi‐isotropic stacking),^[^ ^28^ ^]^ our goal here was to select a stacking sequence that has a balanced strengthening of mechanical properties without imposing any specific restrictions on the stacking arrangement. Therefore, we established a design problem with a variable stacked‐angle sequence, defined as (*X*_1_°, *X*_2_°, *X*_3_°, *X*_4_°), for a four‐layer stacked mat.

A machine learning‐based optimization method was applied to identify various stacking sequences of a four‐layer stacked mat configuration with the aim of tailoring and enhancing the target mechanical properties of multistacked yarns. As addressed in the Introduction, to the best of our knowledge, there are no reports to date on optimal stacking angle sequences for strengthening multistacked electrospun piezoelectric yarns. In material design, to strengthen materials, it is often necessary to simultaneously consider various mechanical characteristics with tradeoffs or conflicting correlations, which can lead to challenges with a large design space, especially for materials with high experimental costs.^[^ ^29^ ^]^ In the case of piezoelectric yarns, our focus was on the following two mechanical properties: UTS and failure strain. These properties are major factors in determining toughness and are observed to have a tradeoff relationship, as shown in Figure 3e,f. Therefore, we introduce a Gaussian process regression (GPR)‐based MBO framework to maximize the two objective functions, the UTS (*f*_1_) and failure strain (*f*_2_), which can be derived from a 4‐layer stacking sequence as design variables. MBO is a well‐established method for a more efficient exploration of the design space to find the maximum (or minimum) point for the targeted material properties.^[^ ^30^ ^]^ Figure 4b presents an overall machine learning flowchart of the MBO framework. For the initial GPR model, we selected a collection of ten different data points as our initial dataset using uniform Latin Hypercube Sampling (LHS), and then experimentally evaluated the values of their objective functions (Figure S16, Supporting Information).^[^ ^31^ ^]^ Uniform Latin Hypercube Sampling is a statistical method used to generate samples of parameter values that are evenly distributed across a multidimensional parameter space. We employed this method to evenly distribute the ten initial data points across the design space to ensure that the training of this data set can better inform the initial GPR about the overall trend in the design space. Regarding the number of initial training data points, we opted to begin the optimization process with just ten initial training data points because our objective functions are expensive to evaluate. Instead of spending excessive time and resources on collecting many training data points in random areas of the design space, we believed it would be more efficient to start using MBO as early as possible because the data we collect will be more effective for optimization.

Then, the GPR model was trained using the initial dataset, and a new data point was sampled and added to the training dataset for the next iteration. The Bayesian optimization process was repeated, and the GPR model was updated until the termination condition was satisfied. The sampling point was selected by maximizing the value of the acquisition function L(x).^[^ ^30a^ ^]^ The expected hypervolume improvement (EHVI) was introduced as the acquisition function to evaluate the improvement in the hypervolume indicator. A comparison of the predicted and experimental values for the 15 iterations and the relationship between the maximum EHVI value from GPR and the MBO iteration is shown in Figure S17 (Supporting Information). This approach gradually expands the dominated region in the output space, effectively improving the Pareto front, which is well‐established with non‐dominated “Rank 1” solutions, indicating that no other solutions can dominate them^[^ ^32^ ^]^ during sequential Bayesian optimization. This framework is inspired by the MBO model applied to a 2‐objective composite design, as proposed by Park et al.^[^ ^33^ ^]^ GPR, our surrogate model, is a statistical regression model that interpolates observed data to estimate the relationship between input and output data. We considered that GPR is the most suitable surrogate model for our optimization problem, especially when compared to alternatives such as Bayesian neural networks (BNN) and Random forests. This is because our observation data are obtained through real experiments, resulting in each data point having a different noise variance. The heteroscedastic GPR model, our surrogate model in this study, can be trained with data that includes noise variances, a capability that is not easily incorporated into BNN or Random forests surrogate models.

However, because of the limitation of the GPR model, which assumes that all input variables are continuous, it struggles to model the input‐output relationship accurately with our discrete variables, which are composed of eight angles {0°, 30°, −30°, 45°, −45°, 60°, −60°, 90°}. Therefore, unlike the previous MBO framework utilized for the design of nacre‐inspired composites,^[^ ^33^ ^]^ we integrate a special kernel metric into the MBO framework, which incorporates the prior transformation of input locations rounded to the nearest integer before computing the covariance.^[^ ^34^ ^]^

Figure 4c and Figure S18a (Supporting Information) show that the overall prediction mean increased as MBO was iteratively performed. Moreover, Figure S18b (Supporting Information) shows a reduction in the prediction variance with MBO iterations. These results indicated an enhancement in the predictive accuracy of the GPR model. Note that we visualize the GPR model's performance in two dimensions, varying only the second and third angles, represented by angle 2 (*X*_2_) for the x‐axis and angle 3 (*X*_3_) for the y‐axis, respectively, in Figure 4c and Figure S18 (Supporting Information), while fixing the first and fourth layer angles such that (−60°, *X*_2_°, *X*_3_°, 90°) because depicting a 4D space for four variables is not feasible. Additionally, Figure S19 (Supporting Information) includes 1D representations of the trained GPR model at different MBO iterations under the condition that the 4th layer angle is the only variable as (−60°, 90°, −60°, *X*_4_°), also confirming the enhanced accuracy of the prediction model with iterations.

In Figure 4d, with the failure strain on the x‐axis and UTS on the y‐axis, 10 initial data points and 25 MBO data points are scattered. Most of the initial data are located in the bottom‐left corner, whereas the MBO data are predominantly distributed toward the upper‐right corner. This trend is further illustrated in Figure 4e, where the bar graph represents the UTS and failure‐strain values for each data point. Despite configurations (90°/90°/90°/90°) and (−60°/0°/−30°/−45°) from the initial dataset showing the highest UTS and the highest failure strain, respectively, they were significantly inferior in terms of the other objective function. However, the MBO data demonstrated an average increase in each objective function and a reduction in the disparity between them, leading to a balanced improvement in both objectives. Finally, the Pareto front is mapped and represented by a dark red line on the plot. Among the Pareto solutions, we selected three final designs: i] high UTS, ii] balanced UTS and failure strain, and iii] high failure strain, all of which exhibited relatively distinct performances in the two objective functions, failure strain and UTS (Figure 4d; Figure S20, Supporting Information). We select the optimal configuration with a stacking sequence of (−60°/90°/−60°/−60°) for our final yarn design, considering its balanced improvement of both UTS and failure strain performance. There was a remarkable improvement over the initial data (the average value of ten cases of initial data), with UTS increasing from 24 to 49 MPa, a 100% increase, and failure strain increasing from 250% to 288%, a 15% increase (Table S1, Supporting Information). The detailed fabrication process for this optimal design is depicted at the top of Figure 4d, along with the tensile test results.

### 2.5 Corona Poling Postprocessing for High‐Performance Piezoelectric Yarns

**Figure 5:**
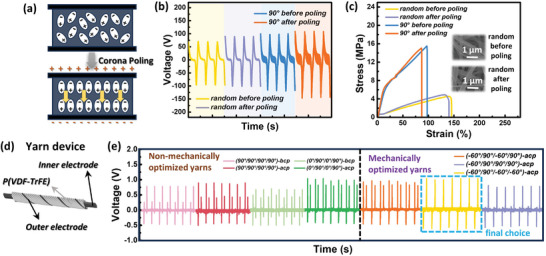
Corona poling postprocessing for piezoelectric yarns. a) Corona poling mechanism. b) Piezoelectric output voltages as a function of time for random and aligned fiber mats before and after corona poling under mechanical bending. c) Stress–strain curves of random and aligned fiber mats before and after corona poling. (Inset: SEM images, magnification: × 6000.) d) Schematic illustration of the yarn device architecture. e) Piezoelectric output voltages as a function of time for (left) two non‐mechanically optimized yarns (90°/90°/90°/90°) and (0°/90°/0°/90°) before and after corona poling (denoted as bcp and acp, respectively), and (right) mechanically optimized yarns (−60°/90°/−60°/90°) & (−60°/90°/−60°/−60°) & (−60°/90°/90°/90°) after corona poling (denoted as acp).

Although thermal annealing is a common postprocessing method for electrospun fibers,^[^ ^10^ ^]^ we observed that heat treatment can compromise the ductility of the fibers owing to increased crystallinity (Figure S21, Supporting Information). Given our objective of achieving dual enhancement in both the piezoelectric and mechanical properties, we decided to omit the annealing process and instead, focused on post‐treatment through corona poling. The principle behind the enhancement of piezoelectric performance with corona poling is dipole alignment for further polarization rather than an increase in crystallinity (**Figure** **5**a). Initially, we optimized the corona poling parameters of the fiber mats, such as the poling voltage, tip‐to‐sample distance, and time (Figure S22, Supporting Information). In conventional methods, corona poling is frequently applied to generate poled fiber mats for yarn production; therefore, the structural twisting of such mats can introduce randomness in the polarization direction, rendering them ineffective in enhancing the piezoelectric performance. As confirmed by the results shown in Figure S23 (Supporting Information), the twisting process disrupted the aligned states of some dipoles. To address this, we devised a system for uniform corona poling of yarns derived from optimized stacked mats, ensuring consistent polarization alignment. Under the influence of the electric field, the dipoles that were slightly twisted during the twisting operation were returned to a completely aligned state, leading to further enhancement in the piezoelectric performance.

Before subjecting the yarn samples to corona poling, we conducted bending and tensile tests on the fiber mats both before and after corona poling, as depicted in Figure 5b,c. The results demonstrate that the corona poling process can enhance the piezoelectric performance without compromising the mechanical properties, thus aligning well with our goal. Furthermore, we characterized the mat and yarn samples before and after corona poling using SEM images (Figure 5c inset, Figure 4‐ii; Figure S24, Supporting Information). The SEM images show that the corona poling treatment had no significant impact on the fiber morphology at the macroscopic level. This observation also explains why the mechanical properties of the fibers did not undergo noticeable changes after corona poling.

To further characterize the piezoelectric performance of the yarn, we conducted a mechanical pushing test to investigate the response of our piezoelectric yarn to various pressure levels. The architecture of the piezoelectric yarn device is shown in Figure 5d: the inner electrode forms a core‐shell structure with the P(VDF‐TrFE) functional layer, and the outer electrode is wound around this core‐shell structure (Figure S25, Supporting Information). Optical microscopy images of the yarn device in Figure S26 (Supporting Information) confirmed good contact between the outer electrode and functional layer. We conducted pushing tests on both the non‐mechanically and mechanically optimized yarns under a load of 30 N. The results in Figure 5e indicate that, regardless of mechanical optimization, the piezoelectric response of the corona‐poled yarns remained at a similar level but was enhanced compared to that of the non‐corona‐poled yarn. The piezoelectric responses of the yarn device at other loads are shown in Figures S27 and S28 (Supporting Information). Even for the three yarns mechanically optimized via MBO, which had the highest UTS [i], balanced UTS and failure strain [ii], and highest failure strain [iii], the piezoelectric performance was maintained at a similar level, as shown in the XRD analysis results in Figure S29 (Supporting Information), despite their different advantages in terms of mechanical performance.

### 2.6 Demonstration of Self‐Powered Yarn Sensing Applications

**Figure 6:**
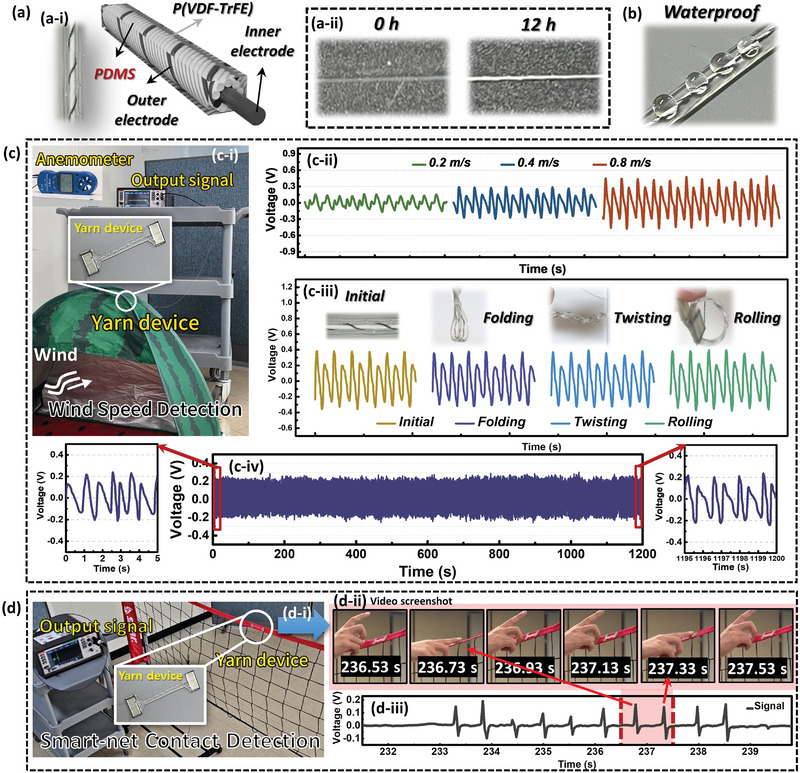
Demonstration of self‐powered flexible yarn sensor applications. a‐i) Illustration of the optimized yarn device (−60°/90°/−60°/−60°) together with a digital photo of the fabricated yarn device. a‐ii) Digital photos of yarns coated with PDMS under different pre‐curing times: for 0 and 12 h. b) Waterproof demonstration of the fabricated yarn device. c) Application of self‐powered real‐time wind monitoring yarn sensor for camping tents: c‐i) experimental setup; c‐ii) output voltage waveforms at different wind velocities; c‐iii) output voltage of the yarn device before and after deformation such as folding, twisting, and rolling, (Inset: Digital photos of yarn device after deformation); and c‐iv) durability test result of the yarn device. d) Application of self‐powered yarn sensor in volleyball competition: d‐i) experimental setup; d‐ii) digital photos of human hands touching the smart net, taken from video screenshots in a time sequence; and d‐iii) output voltage of the yarn device for volleyball smart‐net touch detection.

The self‐powered flexible yarn sensor developed in this study demonstrated practical application in various scenarios, demonstrating its versatility. Two notable applications include wind‐speed sensing in campsites and contact sensing on volleyball smart nets (**Figure** **6**). Building upon the previously optimized yarn device, we introduced an additional protective layer of polydimethylsiloxane (PDMS) coating to enhance its practical applicability. Newly found is that selecting a PDMS solution with an appropriate viscosity is significantly crucial for the coating process. Here, we controlled the solution viscosity and the resulting yarn transparency by altering the pre‐curing time, which is the time from the completion of the PDMS solution preparation to the start of the coating (Figure S30, Supporting Information). If the solution viscosity is too low, for instance, with a pre‐curing time of 0 h, the nonconductive and transparent PDMS solution may flow into the fiber interstices, resulting in a transparent yarn with no piezoelectricity (Figure 6a‐ii, 0 h; Figure S31, Supporting Information). Ultimately, we selected a pre‐curing time of 12 h to preserve the intact morphology of the fiber (Figure 6a‐ii, 12 h). With an appropriate PDMS coating, our yarn device retained high flexibility while also gaining water resistance, as confirmed in Figure 6b. This improvement allows our yarn device to be applied in more challenging environments. The first challenge is the real‐time monitoring of wind conditions during camping. By strategically placing the yarn sensor attached to the tent fabric (Figure 6c‐i), the attached yarn device could detect variations in wind speed and provide valuable data to campers by converting mechanical stimuli from wind into electrical signals (Video S1, Supporting Information). As depicted in Figure 6c‐ii, the yarn device proved to be highly sensitive to wind speed, with the output voltage significantly increasing with the wind speed. We also conducted nonlinear fitting of the wind speed and peak‐to‐peak output voltage to directly infer the wind speed from the output voltage (Figure S32, Supporting Information). Given that tent fabric inevitably deforms when folding and being stored in a bag when not in use, we tested whether the output of the yarn device would weaken after various deformations (folding, twisting, and rolling). Tests were conducted at the same wind speed, and the results showed no significant change in the output level before and after deformation (Figure 6c‐iii), confirming the high flexibility of our yarn device. Furthermore, durability tests were conducted on our yarn device by exposing it to long‐term wind at the same speed. The overall output level of the piezoelectric device did not change significantly, as shown in Figure 6c‐iv. As shown in the two insets, the peak‐to‐peak voltages remained the same for the first and last five seconds of the entire duration, demonstrating the mechanical robustness of our piezoelectric P(VDF‐TrFE) yarn device for practical applications. This application enhances campus safety by alerting users to changes in wind conditions, helping them make informed decisions regarding tent setups and potential risks.

In the second application, a self‐powered yarn sensor served as a contact sensor for volleyball nets. Placing sensor yarns along the net allows the detection of player interactions and ball impacts, providing valuable information for performance analysis, referee decision support, and enhancement of the overall sporting experience. As illustrated in Figure 6d‐i, we integrated our piezoelectric yarn device with a volleyball net to create a smart net. During volleyball games, detecting fouls using cameras or the naked eye can be challenging. However, our smart net can ascertain whether someone has touched the device based on the voltage value (Video S2, Supporting Information). Because of the high sensitivity of our piezoelectric yarn, even a slight pressure contact can generate a voltage (Figure 6d‐ii). Additionally, because our yarn device has good mechanical properties, it can sense pressure even after being hit by a volleyball (Video S3, Supporting Information). To demonstrate this property, we simulated the process of a volleyball striking the net, as shown in Figure S33 (Supporting Information). By combining real‐time photographs with voltage data, it is evident from the voltage output curve that the volleyball contacts the smart net, providing ample evidence for the sensitivity of our yarn device and its potential application in real volleyball matches. A self‐powered yarn sensor is a versatile tool for incorporating smart‐responsive features into sports equipment.

## 3 Conclusion 

In summary, our research presents a novel multilayer stacking strategy, drawing inspiration from the strengthening mechanism found in composite laminates, to enhance the mechanical and piezoelectric properties of P(VDF‐TrFE) yarns derived from electrospinning. By leveraging an MBO‐based machine learning framework, we systematically optimized the stacking sequence of four multilayer electrospun mat layers with subsequent mechanical reinforcement by twisting to yield yarns. The resulting optimized yarn demonstrated a remarkable improvement, with a 153% increase in UTS and a 32% increase in failure strain, compared to prior work involving random fiber yarns (UTS 19.35 MPa and failure strain 218.0%).^[^ ^14^ ^]^ Additionally, the optimized yarn benefits from corona poling, resulting in a 15% increase in *β*‐phase crystallinity, further boosting its piezoelectric performance. Ultimately, the success of our optimized stacked yarn, particularly with the stacking sequence of (−60°/90°/−60°/−60°), was validated through practical applications in a self‐powered wind monitoring tent and a self‐powered touch recognition smart volleyball net. The findings highlight the superior performance of the yarn sensor in complex environments and emphasize the pivotal role of its optimized mechanical and piezoelectric properties.

## 4 Experimental Details

### Materials

All the chemicals were purchased and used directly without further purification. N, N‐Dimethylformamide (DMF) was purchased from Sigma–Aldrich. P(VDF‐TrFE) powder (FC25, VDF: TrFE = 75:25 mol%) was purchased from Piezotech, Arkema, France. Acetone was supplied by Daejung Chemical & Metals Co. Ltd. Polydimethylsiloxane (PDMS) (Silicone Elastomer Kit) (DC‐184 A/B) was purchased from Dow Corning.

### Electrospinning of P(VDF‐TrFE) Fiber Mats

Two distinct electrospinning solutions were prepared. The first solution consisted of 25 wt.% P(VDF‐TrFE) powder dispersed in DMF, whereas the second solution consisted of 17 wt.% P(VDF‐TrFE) powder dispersed in a solvent mixture of acetone and DMF (2:3 wt%). After adding the P(VDF‐TrFE) powder, the solution was stirred overnight at room temperature (23–25 °C) using a magnetic stirrer. The homogeneous P(VDF‐TrFE) solution was loaded into a 10 mL syringe, sealed with a 25‐gauge stainless steel needle (inner diameter: 250 µm, outer diameter: 510 µm), for the electrospinning process. Electrospinning was carried out using a custom‐made electrospinning machine (ESR200RD, Nano NC) at room temperature (23–25 °C) and relative humidity of ≈35% or 45%. The P(VDF‐TrFE) solution was injected at a rate of 20 µL min⁻¹ using a syringe pump, and a high voltage of 18 kV DC was applied to the needle tip. The lateral movement distance of the syringe was 19 cm (for a large‐area fiber mat). The drum collector, with a diameter of 12 cm and a length of 20 cm, covered with aluminum foils, was set to rotate at a constant speed of 100 rpm (i.e., a linear rotating speed of 0.63 m s⁻¹) for random fibers or 1400 rpm (i.e., a linear rotating speed of 8.82 m s⁻¹) for aligned fibers. The tip‐to‐collector distance was 15 cm. The thicknesses of the electrospun mats were controlled by varying the duration of electrospinning. Subsequently, the electrospun fiber mat was transferred to a paper sheet for material characterization and yarn or device fabrication. Thermal annealing was also performed to compare the performances of the as‐spun and annealed mats. A hot plate was used for thermal annealing the samples at 125 °C for 3 h.

### Fabrication of Piezoelectric Yarns

After preparing the large‐area fiber mat, electrospun mats with different fiber orientations were cut from it, as illustrated in Figure 1b‐i. The specific direction definitions are presented in Figure 1b‐ii. Subsequently, four layers of electrospun mats with different fiber orientations were stacked sequentially, and the two ends (two sides of 2 cm) of the electrospun mats were secured together. This stacking step was omitted for the single‐angle yarn. A custom‐made yarn marker was used to twist the stacked electrospun mats. During twisting, one side of the stacked electrospun mat was firmly held by the fixture, whereas the other side was affixed with a rotating jig. The fiber mat was rotated 20 times at a winding speed of 50 rpm,^[^ ^14^ ^]^ forming a stacked piezo‐yarn. Corona poling postprocessing was also performed using corona poling equipment (THEBLUETECH, South Korea) to further enhance the performance of the stacked piezo‐yarns. The tip‐to‐sample surface distance was maintained at 2 cm, and a DC voltage of 6 kV was applied to the needle tip. A hot plate was employed to heat‐treat the samples at 80 °C for 45 min under these conditions.

### Characterizations of Electrospun Fiber Mats and Stacked Yarns

Field‐emission SEM (FESEM, JSM‐7600F, JEOL) at the MEMS·Sensor Platform Center of Sungkyunkwan University (SKKU) was employed to analyze the morphological structures of the electrospun mats and yarns. Before the SEM measurements, all samples were coated with platinum to prevent charge accumulation. The acquired SEM micrographs (at ×1000 magnification) were converted into a grayscale Tagged Image File (TIF) format for fast Fourier transform (FFT) analysis using MATLAB software to quantify the degree of fiber alignment within each mat. An X‐ray diffractometer (SmartLAB, Rigaku) was used to identify the crystalline structures of the electrospun mats. XRD patterns were obtained with Cu radiation operated at 40 kV and 30 mA, using a scanning rate of 0.5° min⁻¹ within the 2θ range of 10°−60°, at a step interval of 0.004°. A high‐resolution X‐ray Diffractometer (HRXRD; SmartLAB, Rigaku) was employed to analyze the yarn. Yarn XRD patterns were acquired using Cu (K_α1_) radiation operated at 45 kV and 200 mA, with a scanning rate of 1.5° min⁻¹ within the 2θ range of 10°−60°, at a step interval of 0.01°. The *β*‐phase crystallinity was further quantified by calculating the ratio of the integrated value, adopting the Lorentz function, following the same method as in the previous work.^[^ ^25^ ^]^ To confirm the chemical bonding structures and quantify the *β*‐phase contents of the fabricated fiber mat specimens, Fourier transform infrared spectrometry (FT‐IR, Frontier, Perkin Elmer) was conducted. To eliminate the transmittance effects caused by the porous structure of the electrospun mat, the attenuated total reflection (ATR) mode was employed using a diamond/KRS‐5 (thallium bromoiodide) crystal with the UATR Accessory. FTIR spectra were obtained with 32 scans at a resolution of 4 cm⁻^1^.

### Mechanical Characterization of Mat and Yarn

The mechanical properties of the electrospun mats and yarns were obtained using a precision universal testing machine (AGX‐20kNVD with a 50 N load cell, SHIMADZU, Japan). Fiber mat and yarn samples were prepared using paper frames and craft glue, following the procedures established in prior work.^[^ ^35^ ^]^ The detailed sample preparation process for the fiber mat sample is illustrated in Figure S34 (Supporting Information). Due to the different fiber orientations in the yarn samples and varying diameters of the different yarn types, the diameters of the yarns were captured and measured using optical microscopy (OM, MSP‐120FPS, Digibird) before each tensile test. All the tensile tests were performed at a tensile speed of 240 mm min^−1^. The S‐S curves were measured for each sample until fracture occurred. To ensure the reliability of the results, each measurement was repeated thrice under identical conditions.

### Machine Learning Optimization Methods

The GPR model predicts a new design by assuming that the given observations and new predictions are correlated by a multivariate Gaussian distribution. In this study, the Matern 5/2 function was used as the covariance function for the GPR model. The optimal hyperparameter values for GPR were determined by a Markov chain Monte Carlo algorithm‐based Bayesian inference to meet the maximum likelihood estimation (MLE) standard. Based on the optimal hyperparameter set and 10 initial training data points, it was possible to characterize a complete GPR, which allowed to make a probabilistic prediction of the objective functions with an unknown design. The computations associated with GPR were implemented in Python using an open‐source Gaussian process library called GPy, developed by the Sheffield machine learning group.^[^ ^36^ ^]^

Fifteen iterations of MBO were performed using the EHVI acquisition function. For single objective optimization, expected improvement (EI) has two key aspects: exploitation, which focuses on areas with high predicted values (μ(*x*)), and exploration, which targets areas of high uncertainty (σ(*x*)). The balance between these aspects helps effectively locate points that can enhance a model's predictability and overall performance. However, in this case, dealing with an MBO problem, the expected value of hypervolume was evaluated, *HV*(*f*(*x*)), which is defined by the volume of the region in the objective space dominated by the solution point. The expected value of the hypervolume was integrated by segmenting the region into multiple rectangles (2D), depending on the dimensions of the output space. More detailed information on the resources used to develop the MBO Python script can be found in the Supporting Information, under section “8. Resources used for the development of MBO python script”.

### Fabrication of Fiber Mat Devices and Stacked Yarn Devices

Fiber mat devices were fabricated and characterized by mechanical bending tests. Initially, electrospun mats were produced on aluminum foil (thickness: 15 µm) and were cut into square pieces measuring 1.5 cm × 1.5 cm. The aluminum foil substrate was used as a bottom electrode, while the top electrode (15 µm thick) was slightly smaller than the mat, measuring 1.4 cm × 1.4 cm. This design prevents the formation of short circuits between the top and bottom electrodes. Finally, conductive tape was used to secure the wires to the top and bottom electrodes. The stacked structure was encapsulated with polyimide (PI) tape, placed onto a substrate, a polyethylene naphthalate (PEN) film, and encapsulated with PI tape. The yarn device differed from the mat device, which features inner and outer electrodes rather than top and bottom electrodes. The inner electrode was introduced during the mat‐stacking process when stacking the yarns. Following the stacking of the second layer, a conductive thread (Silver Fiber) was inserted between the second and third layers, after which the third and fourth layers were added. To prevent coil formation during the twisting process, a silver fiber conductive wire with a smaller diameter (≈0.25 mm) was selected as the inner electrode, as depicted in Figure S25 (Supporting Information). Subsequently, the stacked mat underwent ten rotations at a winding speed of 50 rpm. To attach the outer electrode to the device, the pre‐prepared yarn with the inner electrode was co‐twisted with another conductive wire (stainless steel), which had a diameter of ≈0.4 mm, to create the outer electrode. This co‐twisting process was repeated ten times. For the device eventually employed in the application, a yarn device was first constructed, and then applied a polydimethylsiloxane (PDMS) coating. A liquid PDMS precursor (consisting of a 10:1 mixture of the PDMS monomer and curing agent) was prepared and left at room temperature for 12 h before use. This step was crucial because direct use of the precursor caused the fibers to become transparent, as illustrated in Figure S30 (Supporting Information). Subsequently, the upper surface of the yarn was just submerged in the PDMS solution and then subjected to a drying process in an oven set at 60 °C for 3 h, producing the final yarn sensor.

### Testing of Mats and Stacked Yarn Devices

For the mat device, to assess the piezoelectric output performance of each specimen, measurements were conducted through mechanical bending. A radius‐bending tester (JIRBT‐260, JUNIL TECH) was used to perform repetitive movements. All the mat devices were subjected to consistent strain conditions at a radius of curvature of 5 mm and operated at a frequency of 1 Hz. A digital multimeter (Keithley, DMM6500) was employed as the data acquisition system to record the output voltage across the device under an external load resistance of 1 GΩ. For yarn devices, given their high flexibility and softness, mechanical bending is challenging for inducing strain within the yarn. Therefore, the evaluation of the piezoelectric performance of yarn devices requires a pushing test. The output of the yarn devices was assessed by applying force using a pushing tester (JIPT‐120, JUNIL TECH). Measurements were taken when forces of 10, 20, and 30 N were applied to the device operating at a frequency of 5 Hz. A Tektronix MDO3052 mixed domain oscilloscope with a 40 MΩ input impedance was utilized to determine the voltage output.

### Demonstration of Applications

After creating the yarn sensor, it was attached to a tent to detect wind speed. The wind was manually applied by the experimenter, and wind at the same speed was applied by controlling the angle of the cardboard fan to mimic natural wind conditions. As wind by nature does not always blow uniformly at the same speed, uniform wind waves produced by electric fans were not intentionally used. Specific wind speed was measured using an anemometer (KR‐1880). A digital multimeter (Keithley, DMM6500) was employed as a data acquisition system to record the output voltage. The yarn sensor was then pasted onto the edge of the volleyball net to create a smart net. A digital multimeter (Keithley, DMM6500) was employed as the data acquisition system to record the output voltage and detect whether something was in contact with the smart net.

## Conflict of Interest

The authors declare no conflict of interest.

## Supporting information

Supporting Information

Supplemental Video 1

Supplemental Video 2

Supplemental Video 3

## Data Availability

The data that support the findings of this study are available from the corresponding author upon reasonable request.
