# Supplementary material for: Multi‐Objective Bayesian Optimization for Laminate‐Inspired Mechanically Reinforced Piezoelectric Self‐Powered Sensing Yarns
Source: Adv Sci (Weinh). 2024 Jun 27;11(33):2402440. doi: 10.1002/advs.202402440 (PMC11434127; doi:10.1002/advs.202402440)
Supplement: Supplementary file 1 — Supporting Information [file ADVS-11-2402440-s001.docx]

Supporting Information

**Multi-objective Bayesian optimization for laminate-inspired mechanically reinforced piezoelectric self-powered sensing yarns**

Ziyue Yang†, Kundo Park†, Jisoo Nam, Jaewon Cho, Yong Jun Choi, Yong-Il Kim, Hyeonsoo Kim, Seunghwa Ryu, and Miso Kim*

Z. Yang, J. Nam, J. Cho, Y. J. Choi, H. Kim, M. Kim

Department of Advanced Materials Science and Engineering, Sungkyunkwan University (SKKU), Suwon 16419, Republic of Korea

*E-mail: smilekim@skku.edu (M. Kim)

K. Park, S. Ryu

Department of Mechanical Engineering, Korea Advanced Institute of Science and Technology (KAIST), Daejeon 34141, Republic of Korea

K. Park,

Department of Mechanical Engineering, University of California, Berkeley, CA 94720, USA

Y.-I. Kim

Korea Research Institute of Standards and Science, Daejeon, 34113, Republic of Korea

M. Kim

SKKU Institute of Energy Science and Technology (SIEST), Sungkyunkwan University (SKKU), Suwon 16419, South Korea

† These authors contributed equally to this work.

1. Laminate-inspired Multistacked Piezoelectric Yarns

Due to the random orientation, random fibers exhibit similar mechanical properties in different directions, as shown in Figure S1a and S1b. We conduct tensile tests on square-shaped random fiber mat samples in different directions to ensure unbiased mechanical testing, as the square shape prevents the sample geometry from affecting fiber mat mechanical performance. Slight variations arise because, even at very low speeds, the drum collector induces airflow due to its rotation, thereby causing the fibers to exhibit a very low alignment degree. Therefore, from a mechanical performance perspective, we can consider random fiber mats as "isotropic materials." In contrast, for aligned fibers, also tested using square specimens, significant differences are evident in the test results in different directions (Figure S1c and S1d). Similarly, from a mechanical performance standpoint, we can consider aligned fibers as "anisotropic materials."


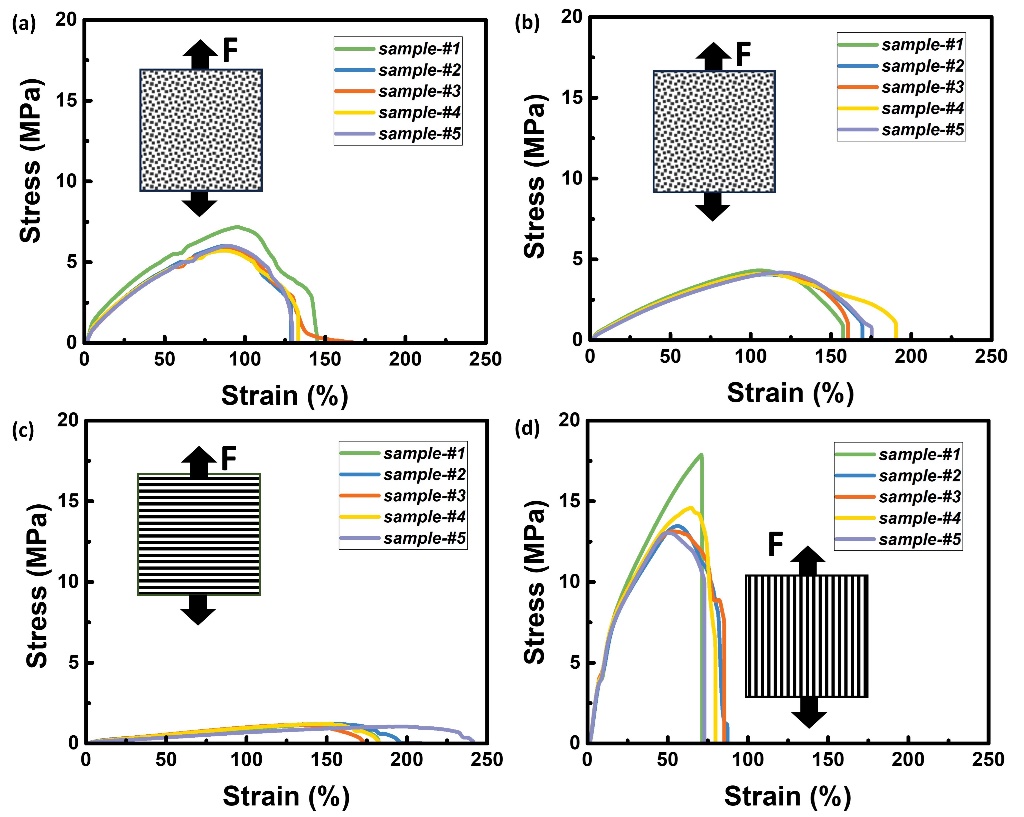


Figure S1. Stress-strain curves of random fiber mat and aligned fiber exert tensile forces in different directions. (a), (b) random fiber mat. (c), (d) aligned fiber mat.

During the experimental process, we also observe that humidity variations during electrospinning affect the alignment degree of fibers (Figure S2, S3). As shown in Figure S2, 45 % relative humidity significantly impacts fiber alignment degree regardless of the solvent or collector material used, while 35 % relative humidity results in a visibly higher alignment degree discernible to the naked eye. With increasing relative humidity, the charge density of the polymer jet decreases, leading to reduced instability and whipping speed. At 35 % relative humidity, both rotation speed and whipping speed showed greater consistency compared to 45 % relative humidity, resulting in improved alignment. To further visualize the alignment degree, we perform image fast Fourier transform (FFT) on four SEM images at 35 % relative humidity (Figure S3). This analysis aims to compare the alignment degree of fiber products obtained under four different parameters. The results indicate that when using DMF as the solvent and aluminum as the collector material, the produced fiber mat exhibits the highest alignment degree (with the least fiber content at angles other than 0° and 180°).


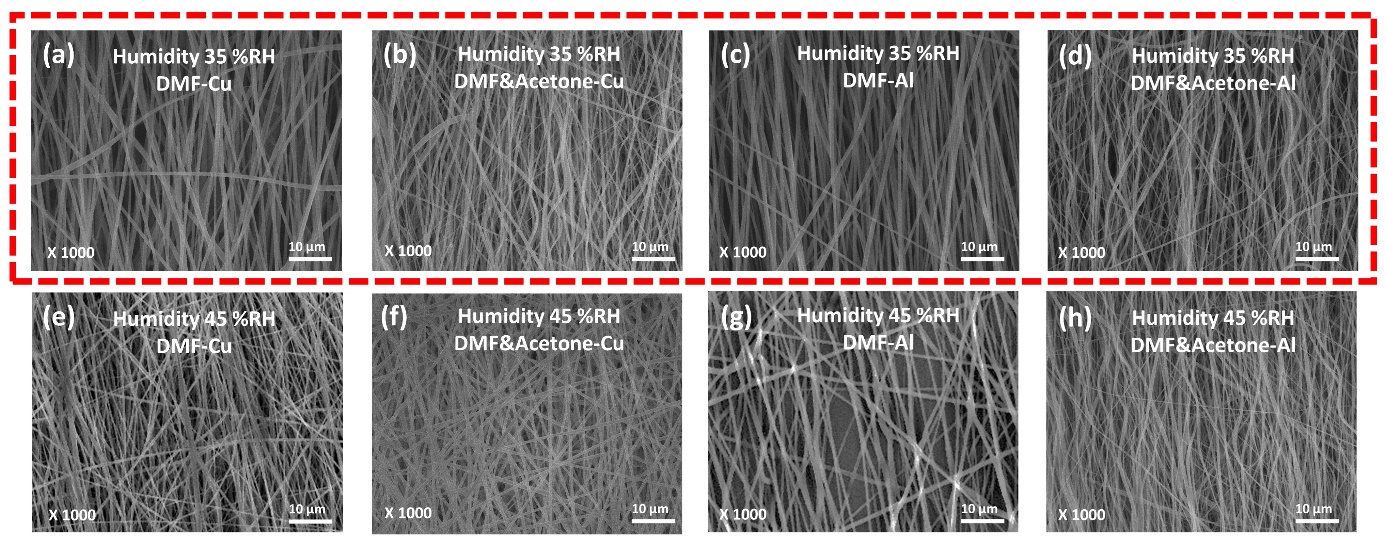


Figure S2. SEM images of fiber mat electrospun under different humidity conditions, using different solvents, and using collectors of different materials. (a) Humidity 35 % RH-DMF-Cu. (b) Humidity 35 % RH-DMF and Acetone-Cu. (c) Humidity 35 % RH-DMF-Al. (d) Humidity 35 % RH-DMF and Acetone-Cu. (e) Humidity 45 %RH-DMF-Cu. (f) Humidity 45 % RH-DMF and Acetone-Cu. (g) Humidity 45 % RH-DMF-Al. (h) Humidity 45 % RH-DMF and Acetone-Cu (magnification: × 1000).


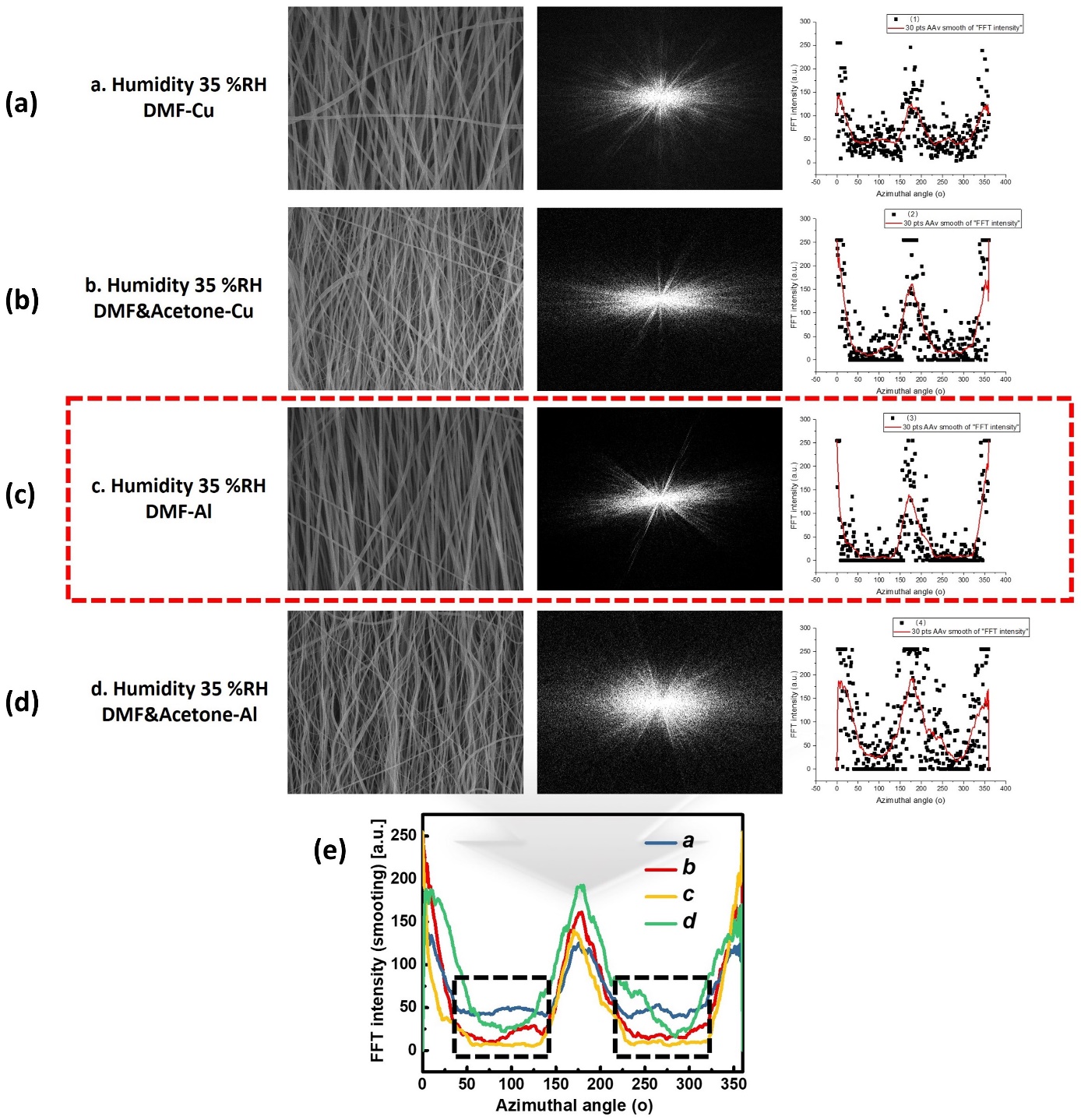


Figure S3. FFT output images and radial intensity distribution vs. detection angle of the fiber mats electrospun under different conditions, (a) Humidity 35 % RH-DMF-Cu. (b) Humidity 35 % RH-DMF and Acetone-Cu. (c) Humidity 35 % RH-DMF-Al. (d) Humidity 35 % RH-DMF and Acetone-Cu. (e) Radial intensity distribution vs. detection angle of the fiber mats electrospun under (a), (b), (c), and (d) conditions.

The use of different solvents can also impact the piezoelectric performance of fiber mats. This is because the differing solvent evaporation rates can influence the formation of the *β*-phase in the fibers. As shown in (Figure S4), we conduct bending tests on two types of fiber mats using DMF, DMF and Acetone (Acetone: DMF = 2:3 wt%) solvents. The results indicate that, at the same thickness, fibers obtained using only DMF as the solvent exhibit a higher output voltage. This is because, compared to the DMF and acetone solvent, DMF has a lower evaporation rate. A low evaporation rate allows sufficient time for the thermodynamically stable *β*-phase to nucleate and grow during fiber formation. This result is consistent with our previous report,^[1]^ where we studied the effect of solvent types and concentration on the piezoelectric performance of fiber devices.


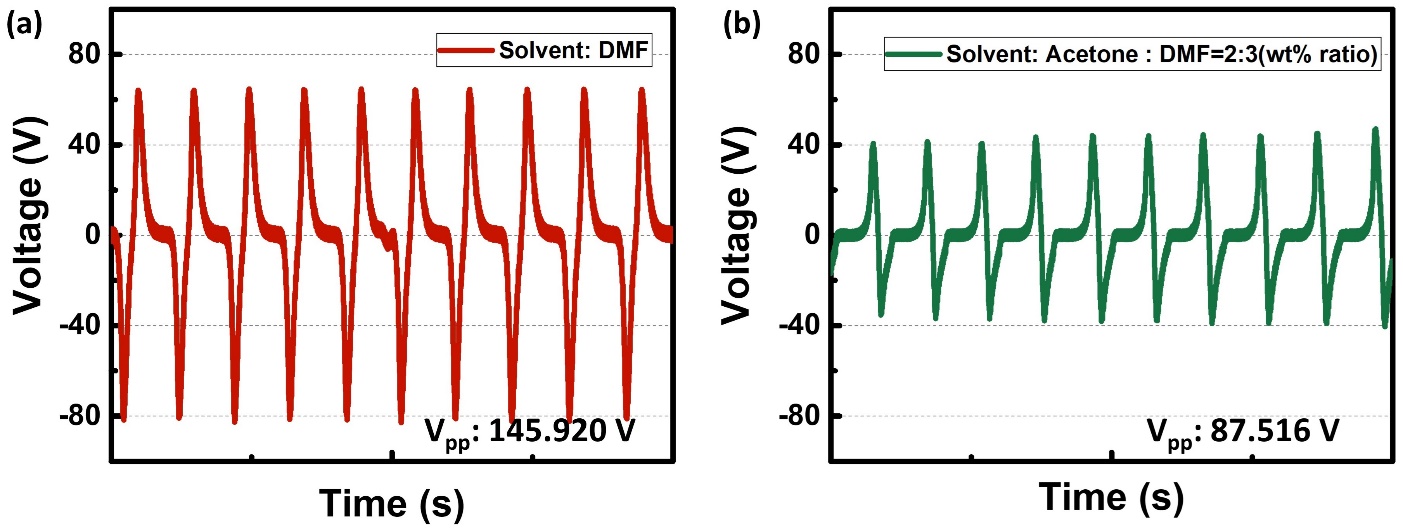


Figure S4. Piezoelectric voltage performance of (a) 25 wt% P(VDF-TrFE) 75/25 mol%(DMF) (thickness: 40 μm). (b) 17 wt% P(VDF-TrFE) 75/25 mol% (Acetone:DMF=2:3(wt% ratio)) (thickness: 40 μm). (frequency: 1 Hz, load resistance: 1 GΩ).

Next, to determine the optimal electrospinning parameters when using DMF as the solvent, we perform electrospinning using solutions of different concentrations, as illustrated in Figure S5. Initially, we obtained uniform diameter fibers at a P(VDF-TrFE) concentration of 25wt%. However, the viscosity of the 25 wt% solution is slightly high. Therefore, we attempt solutions with 22 wt% and 24 wt% concentrations, as shown in Figure S5d, e. Even with a 24 wt% solution, the fibers still exhibit uneven diameters. Consequently, we finalize the electrospinning solution as a 25 wt% P(VDF-TrFE) solution in DMF.


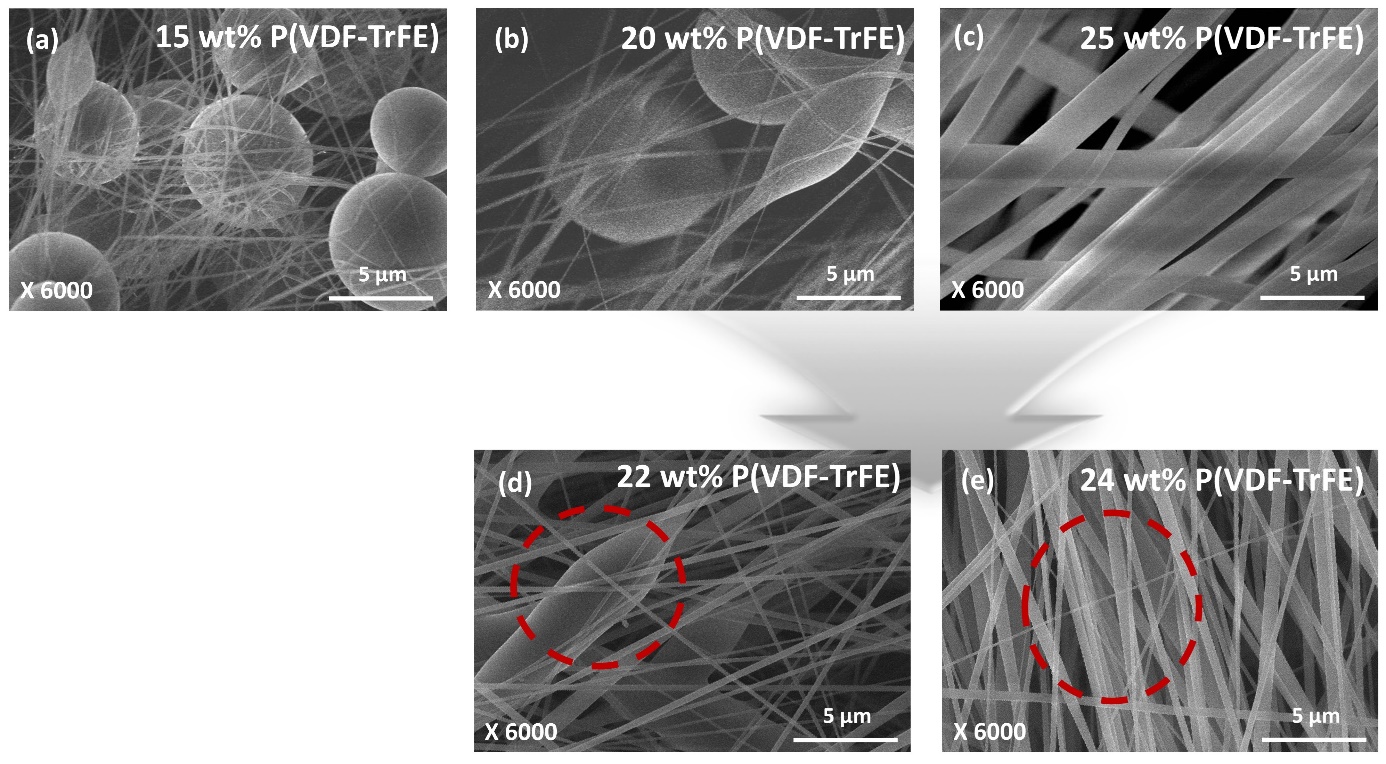


Figure S5. SEM images of morphological evolution with the change of polymer concentration in DMF solvent (a) 15 wt% P(VDF-TrFE). (b) 20 wt% P(VDF-TrFE). (c) 25 wt% P(VDF-TrFE). (d) 22 wt% P(VDF-TrFE). (e) 24 wt% P(VDF-TrFE). (magnification: × 6000).

To investigate whether long-distance lateral movement affects fiber alignment, we longitudinally select four points on the obtained large-area fiber mat for SEM characterization, as shown in (Figure S6). The results indicate that each position's fibers exhibit a similar high alignment degree, confirming that this operation does not impact fiber alignment degree. Simultaneously, we measure the thickness of the fiber mat, as shown in Figure S6c. Due to the influence of electric field forces during the electrospinning process, fibers do not fall vertically onto the collector, resulting in low thickness at both ends of the fiber mat. The central 7.5 cm width of our fiber mat exhibited relatively uniform thickness (approximately 20 μm), sufficient for us to cut fiber mats in different directions. Moreover, we calculate the diameter distribution of the fibers used in our work, as illustrated in Figure S7, showing that our fiber diameters are concentrated around 500 nm.


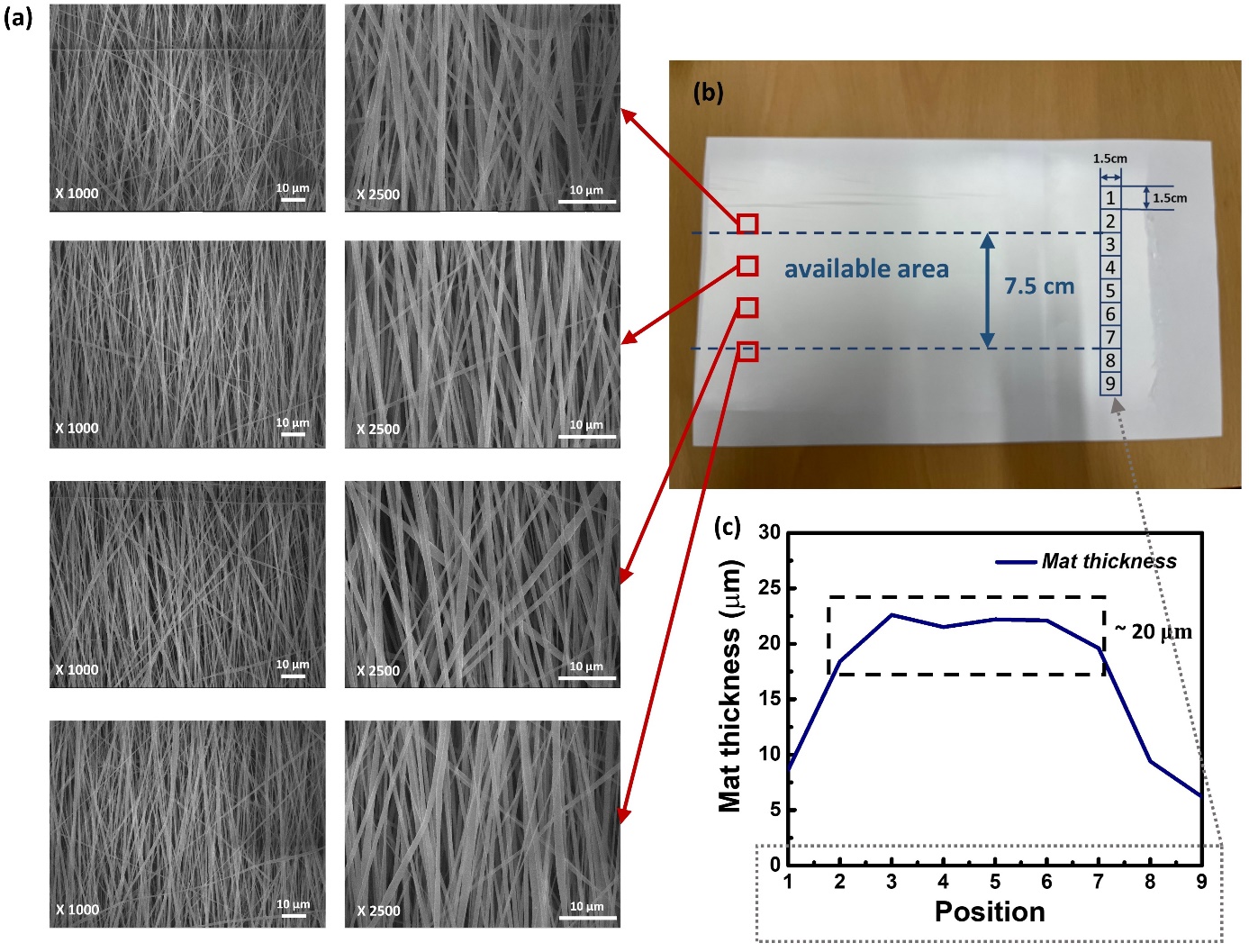


**Figure S6.** (a) SEM images of different parts of the large area fiber mat (magnification: × 1000, × 2500). (b) Digital photo of large area fiber mat. (c) Thickness of different parts of the large area fiber mat.


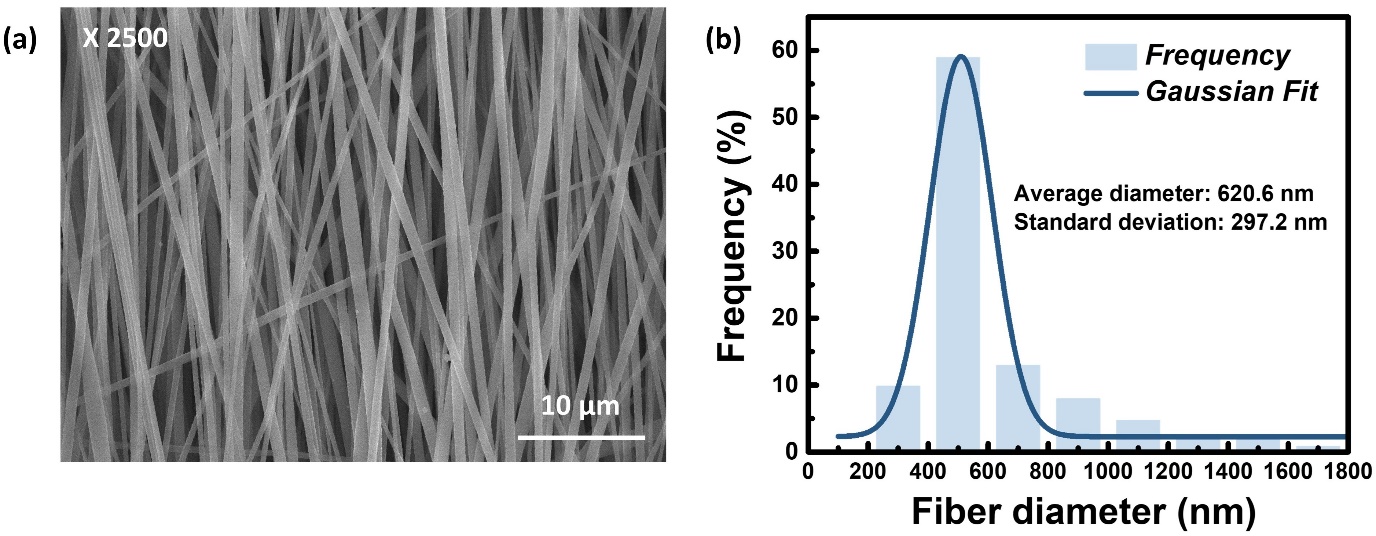


**Figure S7.** SEM image (a) and its fiber diameter distribution (b) of large area electrospun mat.

Inspired by composite laminates, we proceed to stack fiber mats with different orientations to achieve a comprehensive enhancement of mechanical performance. To investigate whether the stacking operation itself affects the performance of fiber mats, we manufacture single-layer and multilayer fiber mats with the same thickness. First, we explore whether it has an impact on piezoelectric performance, as shown in Figure S8. The bending test output voltages for both single-layer and multilayer fiber mats remain at similar levels, confirming that the stacking operation does not affect piezoelectric performance. We test yarn samples to characterize mechanical performance, as stacking alone is insufficient to establish effective interactions between layers for mechanical performance. Twisting operations are needed to connect the layers tightly. The characterization results for the mechanical performance of single-layer and multilayer yarns are shown in Figure S9. We compare three sets of samples: random yarns, horizontally arranged, and vertically arranged yarns. The results indicate that regardless of fiber orientation, stacking has minimal impact on mechanical performance but may have a slight weakening effect on failure strain. Overall, the impact is minimal.


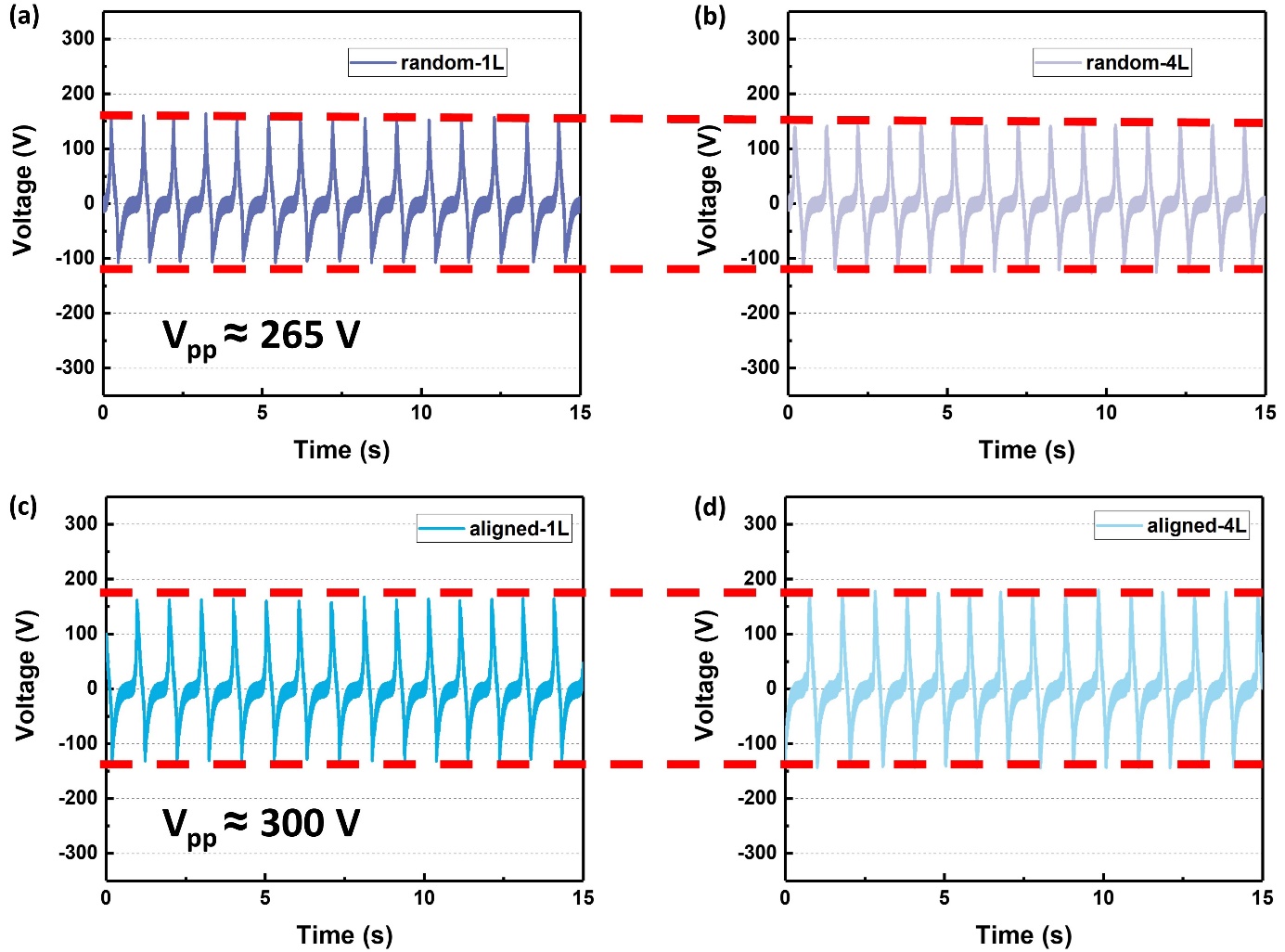


**Figure S8.** Piezoelectric voltage performance of (a) random-1-layer mat (thickness: 80 μm). (b) random-4-layer mat (thickness: 20×4=80 μm). (c) aligned-1-layer mat (thickness: 80 μm). (d) aligned-4-layer mat (thickness: 20x4=80 μm). (frequency: 1 Hz, load resistance: 1 GΩ).


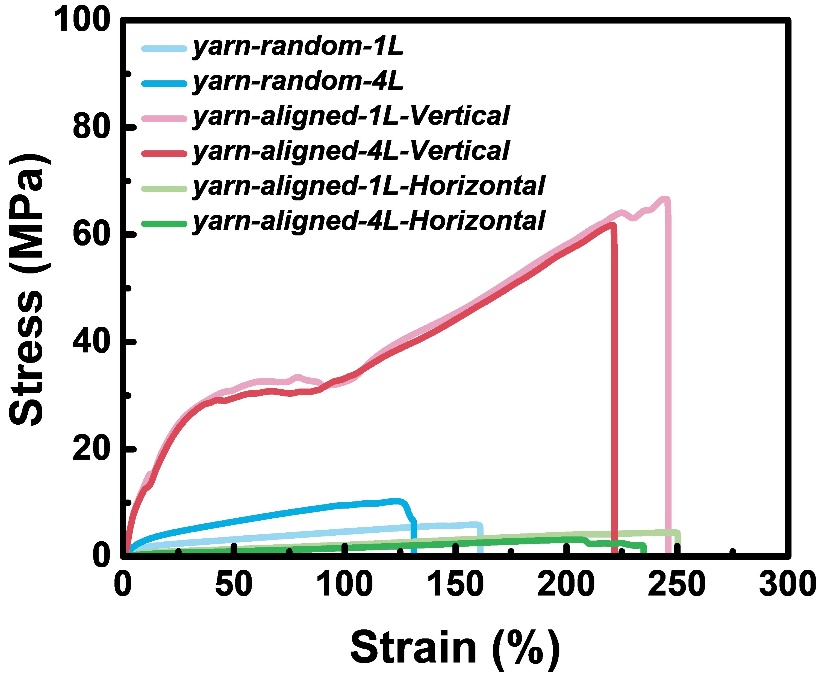


**Figure S9.** Stress vs. Strain curve of 1-layer and 4-layer yarn.

**2. Piezoelectric and Mechanical Characterization of Fiber Mats**


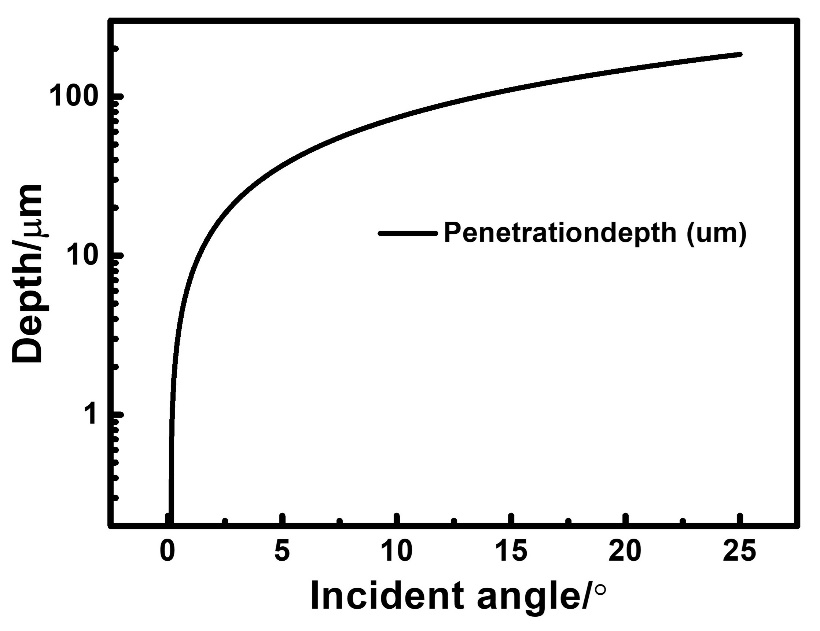


**Figure S10.** The theoretical X-ray penetration depth of P(VDF-TrFE)(75:25) as a function of the incident angle.

The theoretical X-ray penetration depth of the incident X-ray beam, *τ*(α), is given by the following equation:

$\tau(\alpha)=\frac{\lambda}{4\pi\times2^{-1/2}}\left( \sqrt{(\alpha^{2}-\alpha_{c}^{2})^{2}+4\beta^{2}}+\alpha^{2}-\alpha_{c}^{2} \right)^{\frac{1}{2}}$ (1)

where *λ* is the X-ray wavelength (Cu*K*_α_, *λ* = 1.5405 Å), *β* = *μλ*/4π (*μ* is the mass attenuation coefficient), and the critical angle, *α*_c_, is given by

$\alpha_{c}=\text{co}\text{s}^{-1}(1-\delta)$ (2)

In equation (2), *δ* is the real part of the complex refractive index and is defined as

$\delta=\frac{Ne^{2}\lambda^{2}}{4\pi\varepsilon_{o}\left( 2\pi mc^{2} \right)}$ (3)

where *ε*_o_ is the permittivity of vacuum (*ε*_o_ = 8.85×10^-12^ C^2^/*N*m^2^), *c* is the velocity of light, *e* and *m* are the charge and mass of the electron, respectively, and *N* is the number of electrons per unit volume irradiated.

For P(VDF-TrFE) (75:25),

$N=\frac{\rho N_{av}\times\left[ a\left( Z_{C}+\Delta f_{C}^{'} \right)+b\left( Z_{H}+\Delta f_{H}^{'} \right)+c\left( Z_{F}+\Delta f_{F}^{'} \right) \right]}{M}$ (4)

where *ρ* is the density, *N*_av_ is the Avogadro number, *Z* is the atomic number, *M* is the molecular weight (kg/mole), and Δ*f*’ is the real part of the dispersion correction to the scattering factor.

The parameters such as the X-ray wavelength (Cu*K*_α_, *λ* = 1.5405 Å), the average density for P(VDF-TrFE) (75:25) (*ρ*_P(VDF-TrFE)_ = 1.83 g/cm^3^), the critical angle of P(VDF-TrFE) (75:25) (*α*_c_P(VDF-TrFE)_ = 0.138°), and the linear absorption coefficients of P(VDF-TrFE) (75:25) (*μ*_P(VDF-TrFE)_ = 23.701 cm^-1^) for Cu*K*_α_ radiation were used to calculate the theoretical X-ray penetration depth of P(VDF-TrFE) (75:25).


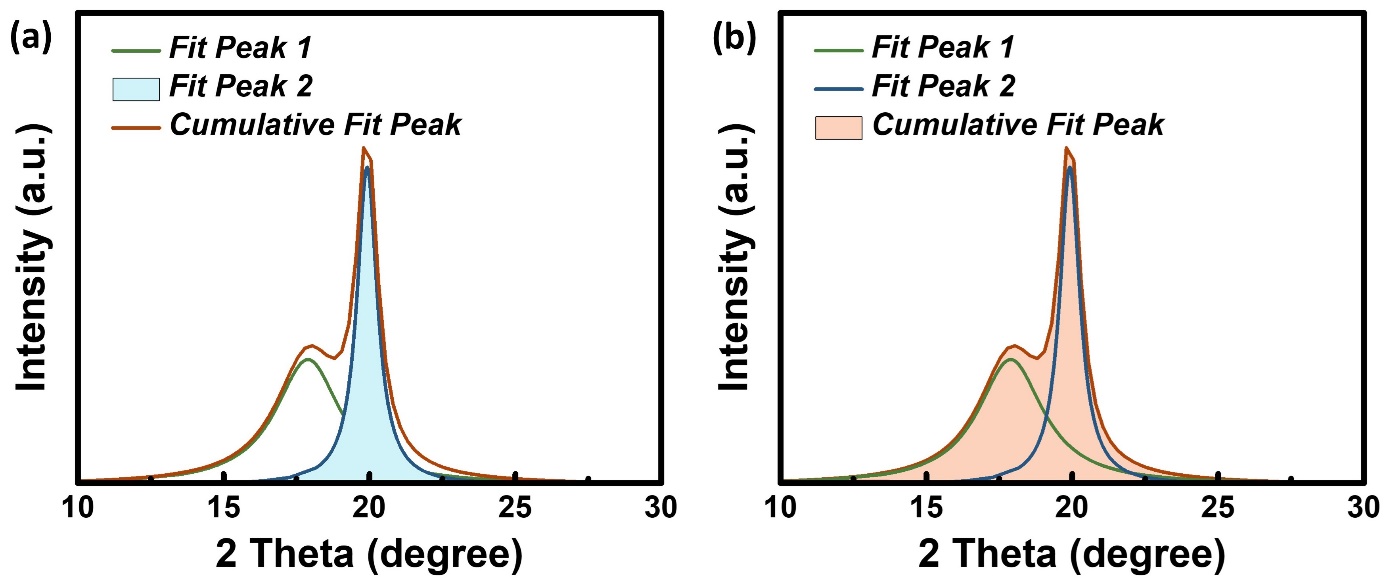


**Figure S11**. XRD data after fitting (using Lorentz function), (a) Area of *β*-phase after fitting, (b) Area of amorphous phase and crystalline phase after fitting.


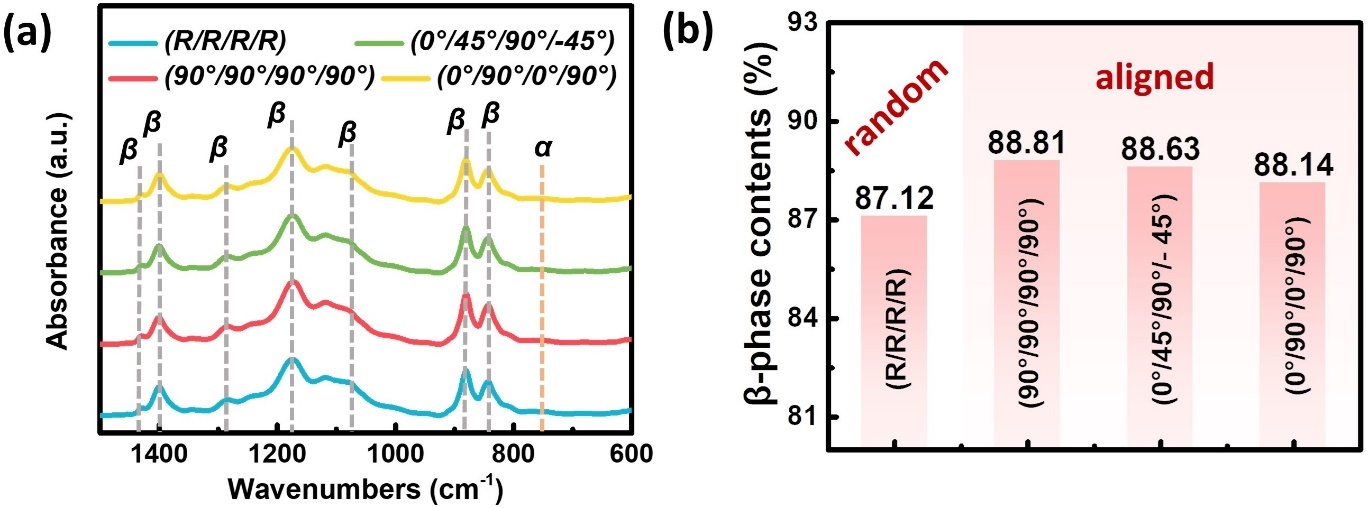


**Figure S12**. (a) FT-IR spectrum of random-one layer (R/R/R/R), aligned-one layer (90°/90°/90°/90°), stacking at different angles-four-layer (0°/45°/90°/-45°) and (0°/90°/0°/90°) fiber mat. (b) *β*-phase contents calculated by FT-IR spectrum.

3. Fabrication and Characterizations of Piezoelectric Yarns


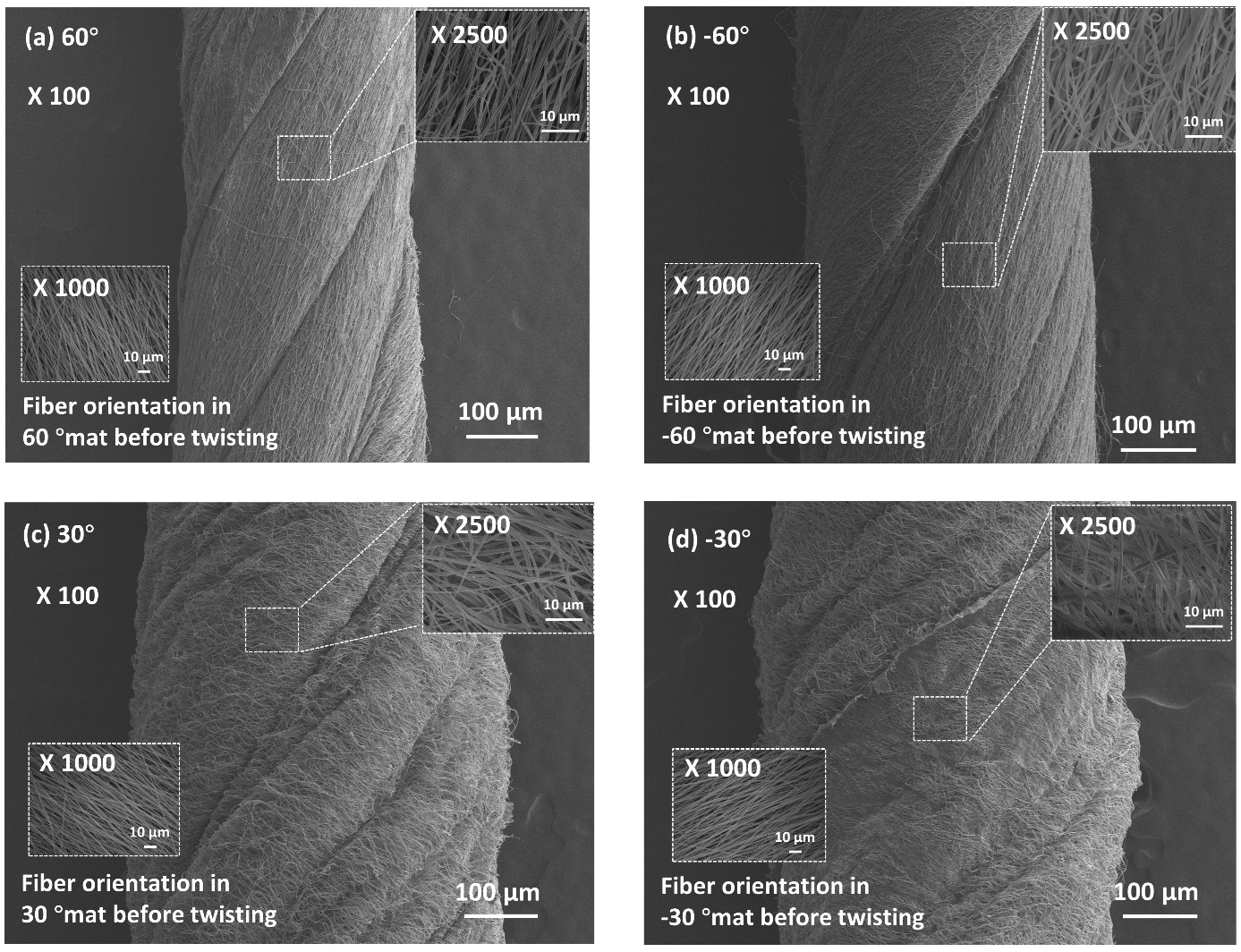


**Figure S13.** SEM images of yarns with different angles of fiber orientation. (a) 60° (b) -60° (c) 30° (d) -30° magnification: × 100, Inset top right: magnification: × 2500, fiber orientation within the yarn. Inset bottom left: magnification: × 1000, fiber orientation in fiber mat (before yarn manufacturing).


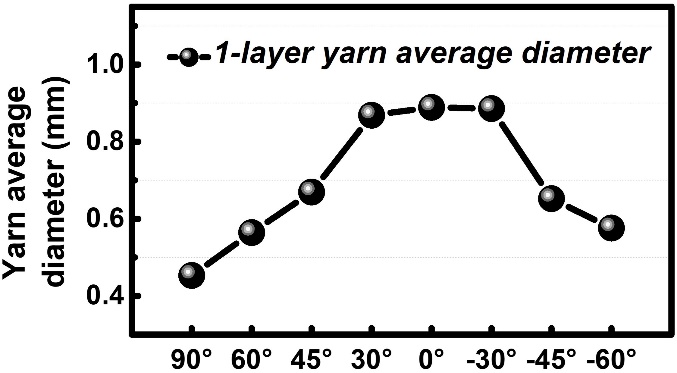


**Figure S14.** The average diameter of the yarn at different fiber orientation angles.

**4. Machine Learning-Based Optimization of Mechanical Performance for Multilayer Stacked Yarns**


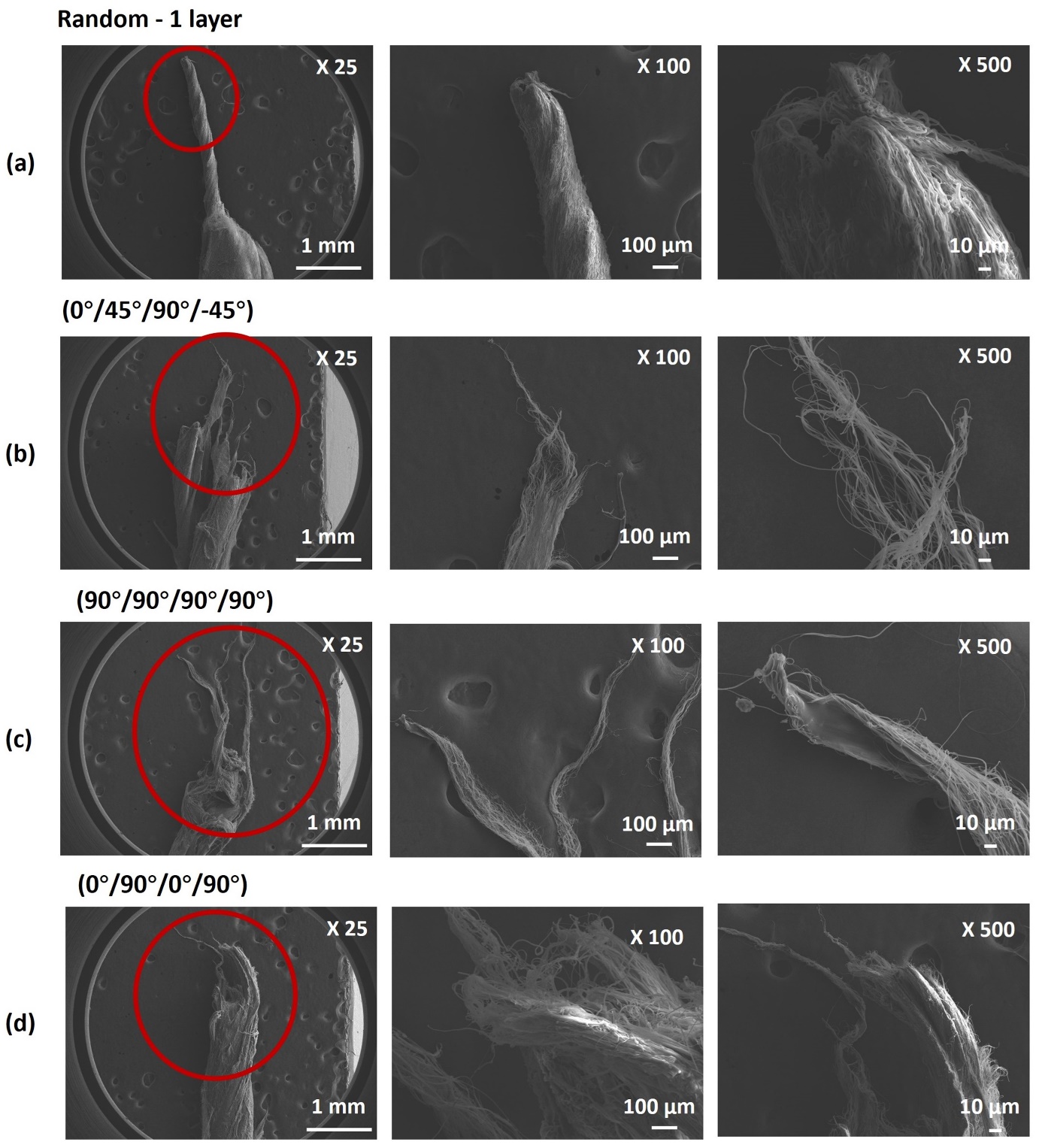


**Figure S15.** SEM images of the cut plane of yarn after failure. (a) Random-1 layer. (b) (0°/45°/90°/-45°). (c) (90°/90°/90°/90°). (d) (0°/90°/0°/90°). (Magnification: × 25, × 100, × 500.)


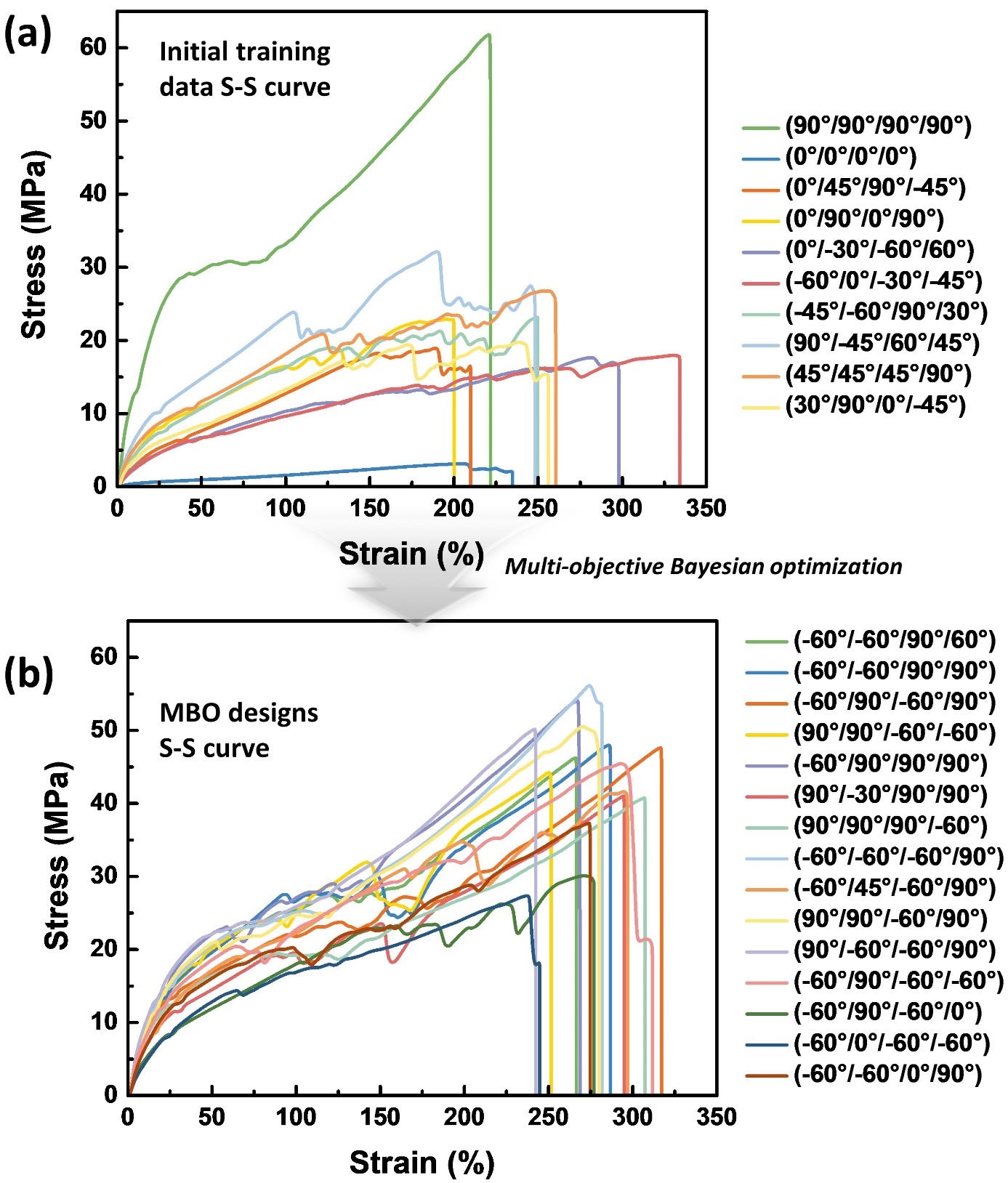


**Figure S16.** Stress vs. Strain curve of yarn (a) Initial training data. (b) Multi-objective Bayesian Optimization (MBO) design data.


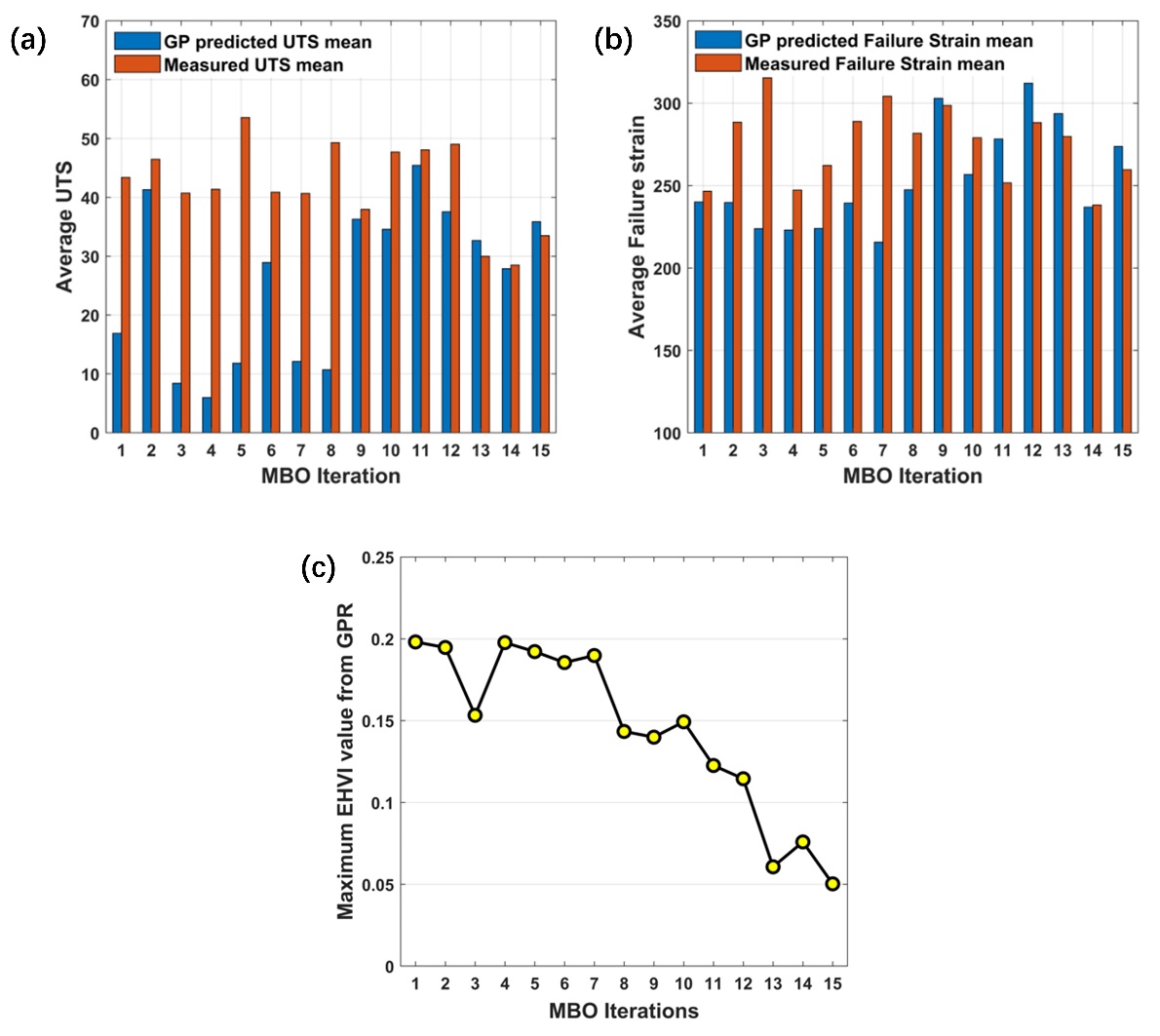


**Figure S17.** The comparison of the predicted and experimental values (a) UTS, (b) failure strain for the 15 iterations, and (c) the relationship between the maximum EHVI value from GPR and the MBO iterations.


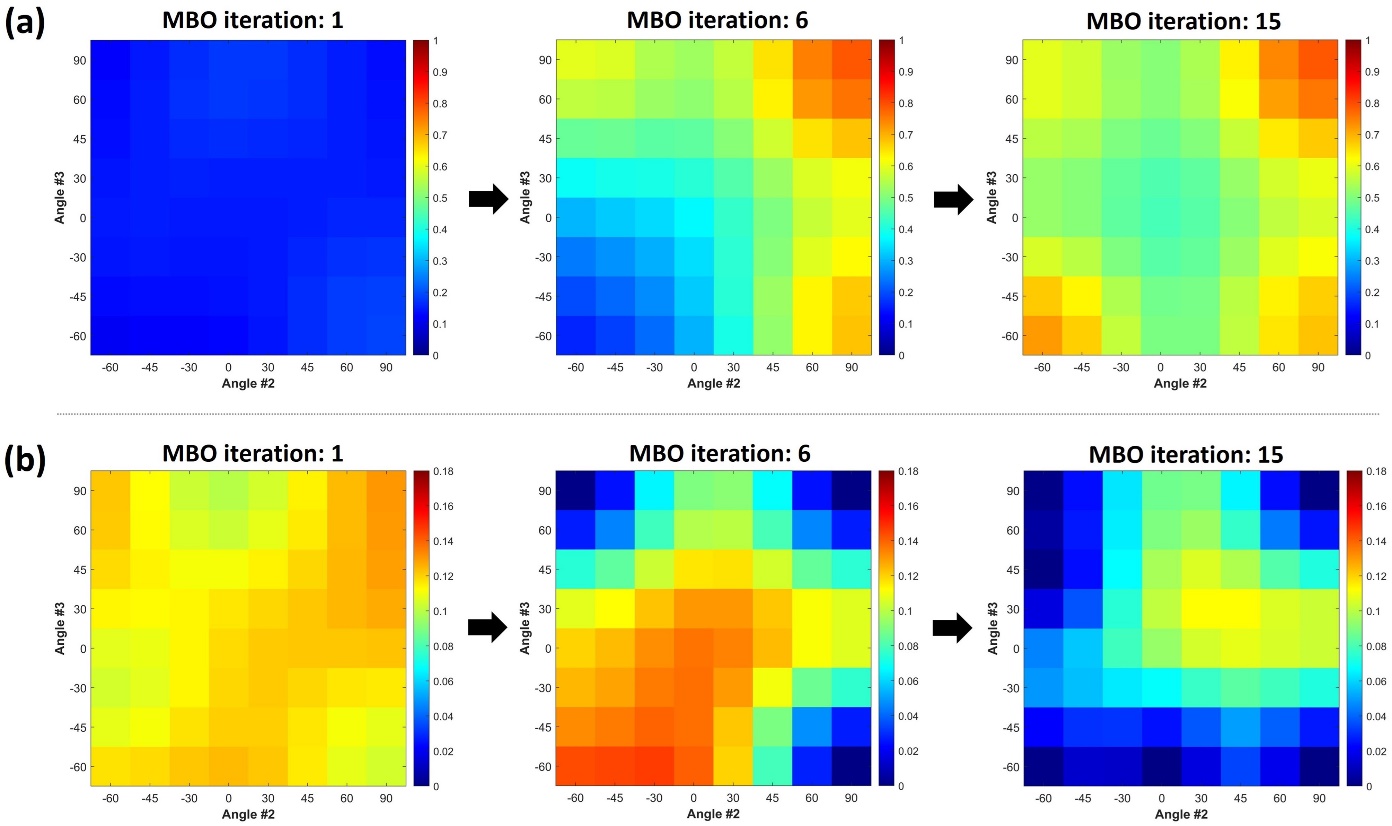


**Figure S18. 2D representation of the trained GPR model for ultimate strength at different MBO iterations.** The 1^st^ and 4^th^ layer angles are fixed to -60°, and 90°, respectively. (a) GP Prediction mean. (b) GP Prediction variance.


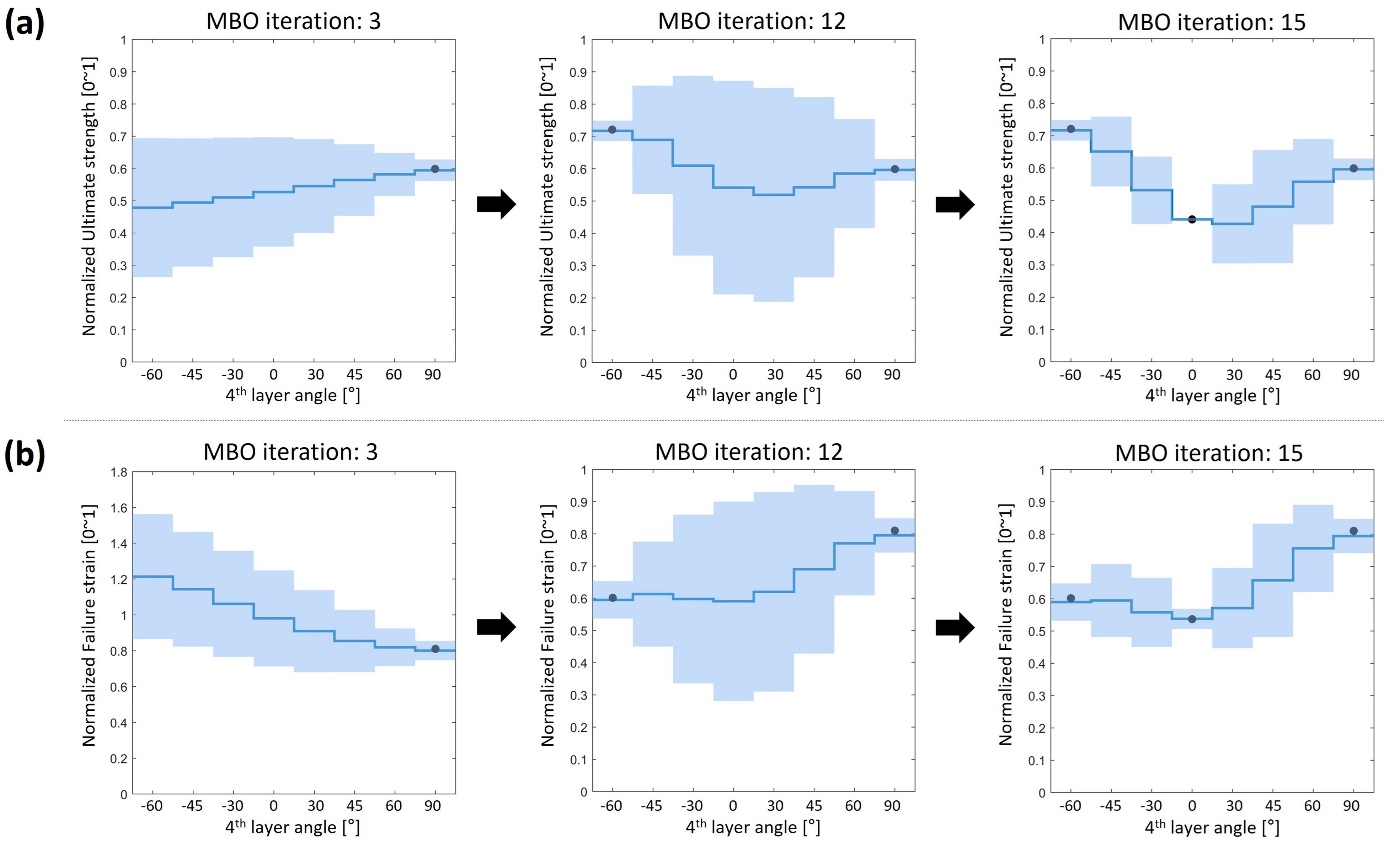


**Figure S19. 1D representation of the trained GPR model at different MBO iterations.**

1^st^, 2^nd^, and 3^rd^ layer angles are fixed to [-60°, 90°, -60°].

The solid line represents the prediction mean, the shaded region represents a 90 % confidence interval, and the dots represent observation data.

(a) GPR model for ultimate strength. (b) GPR model for failure strain.


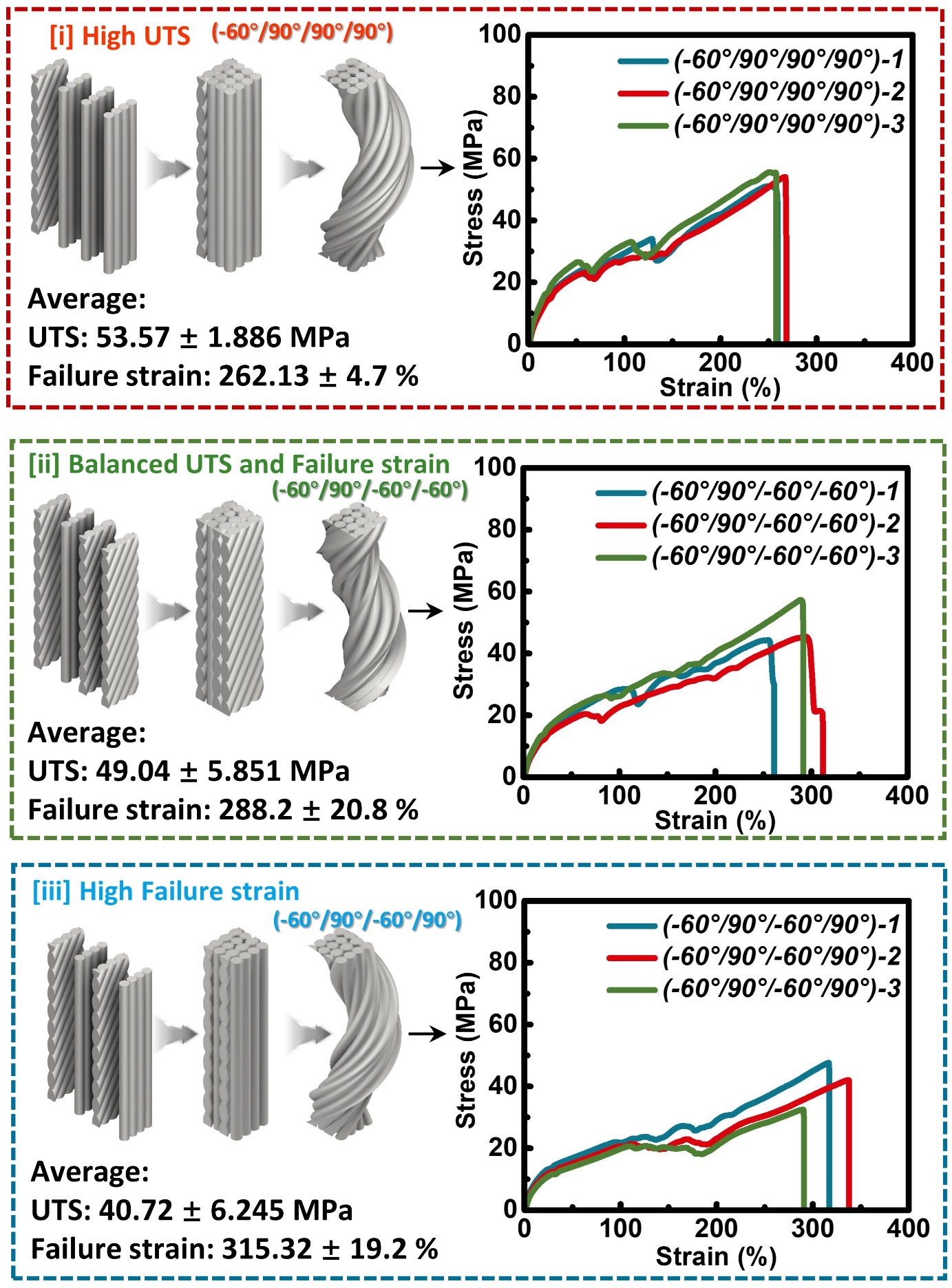


**Figure S20.** The model diagrams and S-S curves of (i) (-60°/90°/90°/90°). (ii) (-60°/90°/-60°/-60°). (iii) (-60°/90°/-60°/90°).

**Table S1.** Table of UTS and failure strain for all data.

| **Stacking sequence** | **Average UTS**  **[MPa]** | **UTS standard deviation** | **Average Failure Strain [%]** | **Failure Strain standard deviation** |
| --- | --- | --- | --- | --- |
| (90°/90°/90°/90°) | 56.65 | 10.88 | 216.00 | 27.04 |
| (0°/0°/0°/0°) | 3.122 | 0.356 | 221.49 | 9.939 |
| (0°/45°/90°/-45°) | 21.17 | 4.902 | 218.64 | 6.512 |
| (0°/90°/0°/90°) | 23.95 | 3.724 | 229.42 | 34.29 |
| (0°/-30°/-60°/60°) | 16.21 | 1.266 | 276.61 | 28.73 |
| (-60°/0°/-30°/-45°) | 18.33 | 0.511 | 333.79 | 38.49 |
| (-45°/-60°/90°/30°) | 24.89 | 1.771 | 251.66 | 8.493 |
| (90°/-45°/60°/45°) | 32.69 | 1.929 | 238.55 | 14.46 |
| (45°/45°/45°/90°) | 26.81 | 1.516 | 258.79 | 2.291 |
| (30°/90°/0°/-45°) | 21.14 | 1.406 | 252.66 | 19.04 |
| (-60°/-60°/90°/60°) | 43.41 | 2.132 | 246.58 | 24.40 |
| (-60°/-60°/90°/90°) | 46.48 | 2.608 | 288.39 | 19.71 |
| **(-60°/90°/-60°/90°)** | **40.72** | **6.245** | **315.32** | **19.21** |
| (90°/90°/-60°/-60°) | 41.38 | 3.241 | 247.21 | 11.35 |
| **(-60°/90°/90°/90°)** | **53.57** | **1.886** | **262.13** | **4.677** |
| (90°/-30°/90°/90°) | 40.88 | 3.314 | 288.89 | 15.24 |
| (90°/90°/90°/-60°) | 40.67 | 2.303 | 304.20 | 10.35 |
| (-60°/-60°/-60°/90°) | 49.28 | 4.854 | 281.63 | 12.86 |
| (-60°/45°/-60°/90°) | 37.97 | 2.585 | 298.61 | 16.48 |
| (90°/90°/-60°/90°) | 47.70 | 4.134 | 279.03 | 6.236 |
| (90°/-60°/-60°/90°) | 48.09 | 2.046 | 251.64 | 15.99 |
| **(-60°/90°/-60°/-60°)** | **49.04** | **5.851** | **288.20** | **20.80** |
| (-60°/90°/-60°/0°) | 29.98 | 0.834 | 279.77 | 11.12 |
| (-60°/0°/-60°/-60°) | 28.50 | 2.855 | 238.28 | 19.20 |
| (-60°/-60°/0°/90°) | 33.50 | 2.882 | 259.58 | 26.02 |

**5. Corona Poling Postprocessing for High-performance Piezoelectric Yarns**

Postprocessing refers to the treatment applied to fibers after their formation, to improve their performance, stability, or meeting specific application requirements. For electrospun fibers, a common postprocessing method is annealing, which involves heat treatment to enhance their piezoelectric properties. However, few studies have explicitly addressed the impact of heat treatment on the mechanical properties of fibers. We conduct tensile tests on fiber mats before and after heat treatment. Figure S21 shows that all other mechanical properties decline except for the ultimate tensile strength (UTS), which remains at a similar level. As shown in the magnified figure inset Figure S21a, the elastic deformation part becomes shorter after annealing. This is attributed to the increase in fiber crystallinity after annealing, resulting in reduced fiber ductility and, consequently, a decline in mechanical performance. Since our ultimate goal is to achieve a dual enhancement in both piezoelectric and mechanical properties, we decided to forgo the annealing process and instead focused our attention on post-treatment through corona poling.


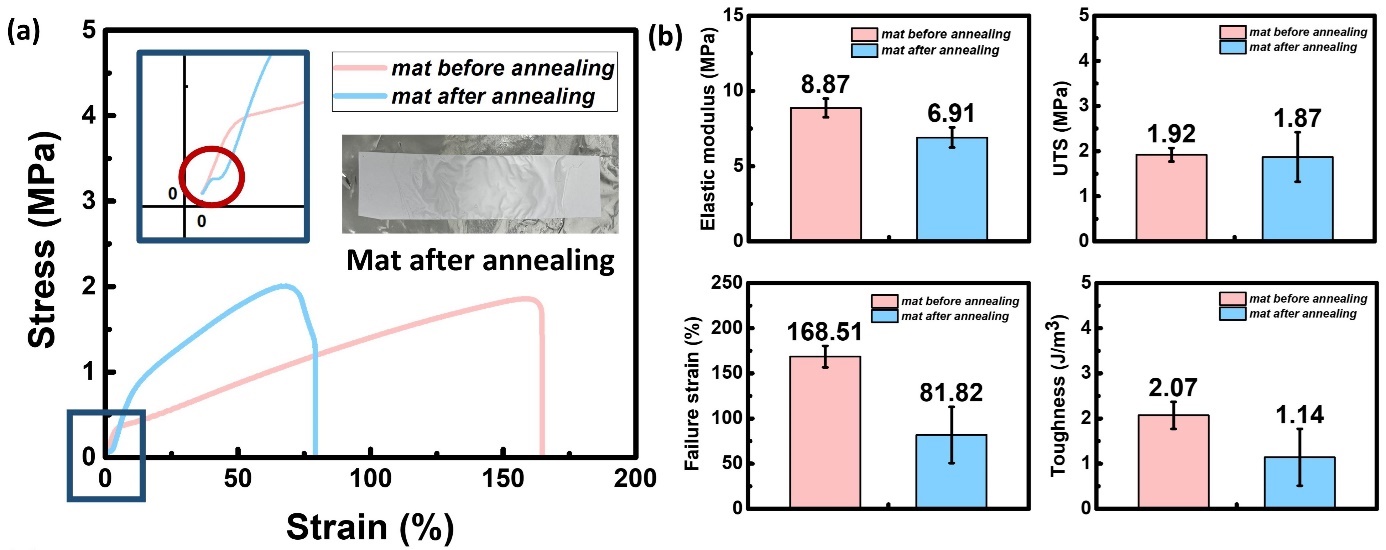


**Figure S21.** Mechanical properties of mat before and after annealing. (a) Stress-strain curves of mat before and after annealing (inset: elastic region of stress-strain curve). (b) Elastic modulus, UTS, failure strain, and toughness of mat before and after annealing.

The impact of corona poling on the piezoelectric performance of the fibers depends on specific conditions. We systematically investigate the effects of polarization voltage, tip-to-sample surface distance, and polarization time on piezoelectric performance, as illustrated in Figure S22. Ultimately, we determine the optimal corona poling conditions, indicating that optimal output can be achieved at 6 kV / 2 cm / 45 min. Subsequent corona poling treatments are conducted under these conditions.


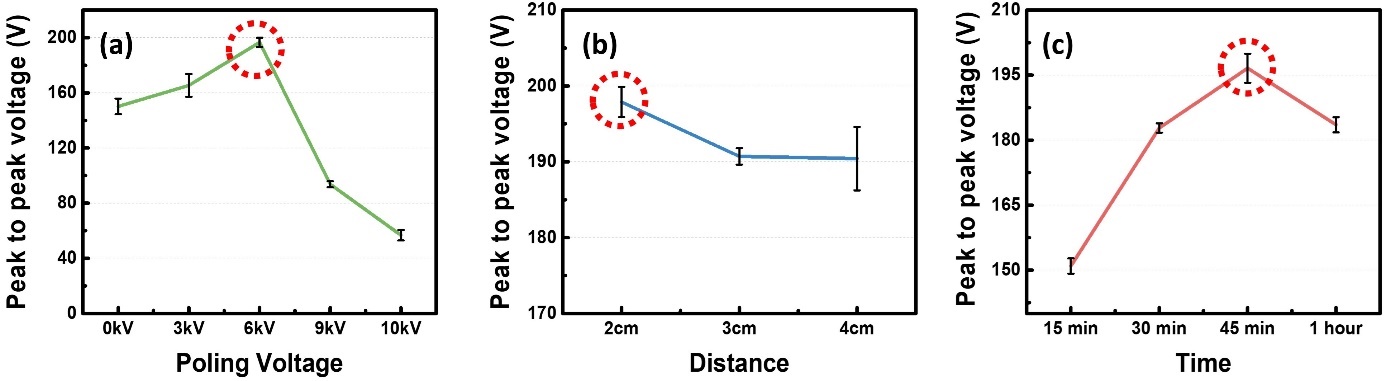


**Figure S22.** The impact of different parameters on the corona poling effect. Relationship between fiber mat bending test peak-to-peak output voltage and (a) poling voltage. (b) tip-to-sample surface distance. (c) poling time. (frequency: 1 Hz, load resistance: 1 GΩ).

As our final product takes the form of yarn, we conduct studies on yarn samples. Two methods of corona poling are explored for yarn samples: one is twisting after poling the fiber mat and the other is first twisting and then poling the yarn. As shown in Figure S23, we explore two methods, and we find that twisting after polarization occasionally improves piezoelectric performance with uneven enhancement levels. This variability is attributed to polarization aligning the dipoles in the fiber mat. Still, twisting may disrupt this alignment degree, leading to inconsistent improvements in the piezoelectric performance of some samples. In summary, we ultimately opt for the method of twisting before poling. The confirmation of this process's effectiveness in enhancing the piezoelectric performance of the yarn is supported by XRD characterization and subsequent calculation of the *β-*phase crystallinity.


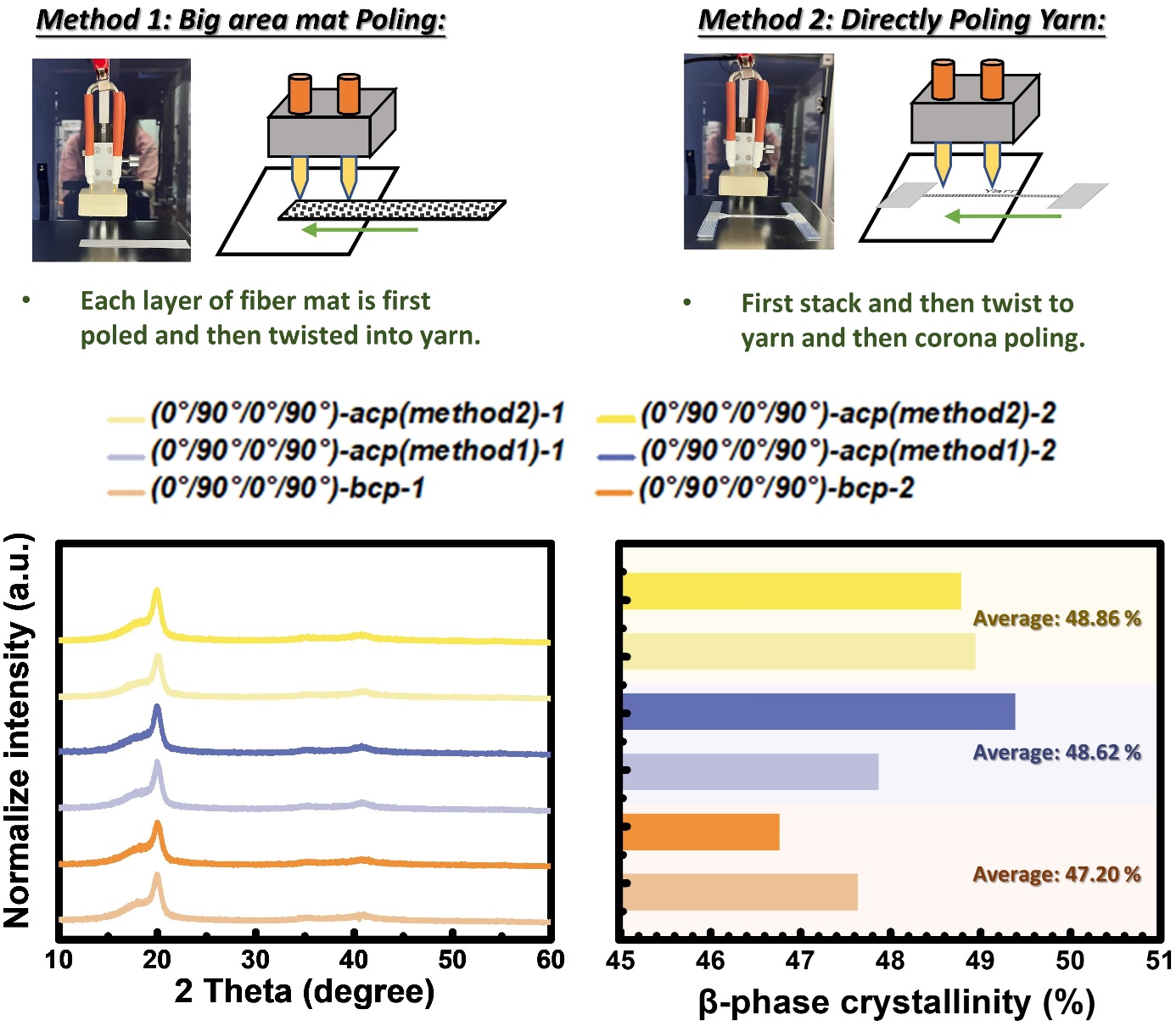


**Figure S23.** XRD comparison of (0°/90°/0°/90°) yarn uses different ways of corona poling (the number of samples per method is two) and *β-*phase crystallinity calculated by XRD measurement.


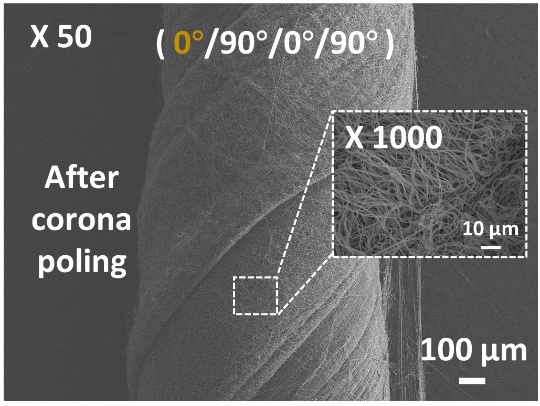


**Figure S24.** SEM images of multistacked yarns (0°/90°/0°/90°) after corona poling. (Magnification: × 50, Inset Magnification: × 1000.)


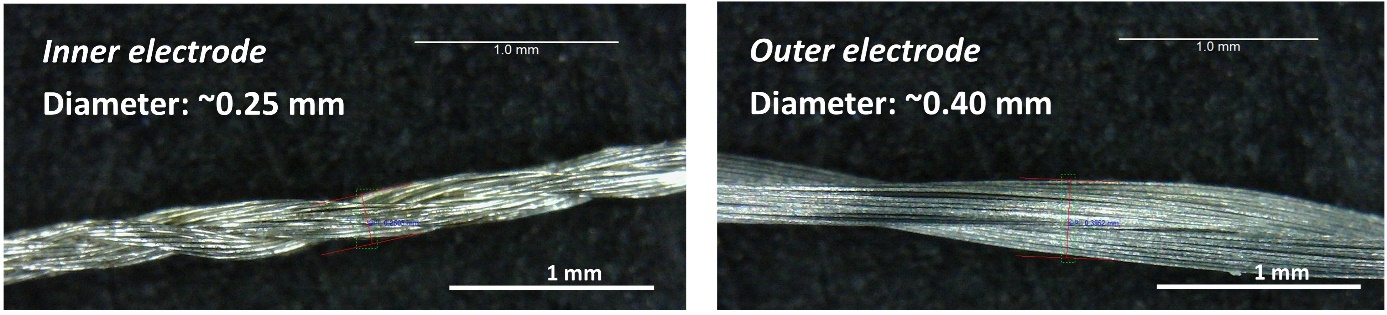


**Figure S25.** OM images of the inner and outer electrodes.


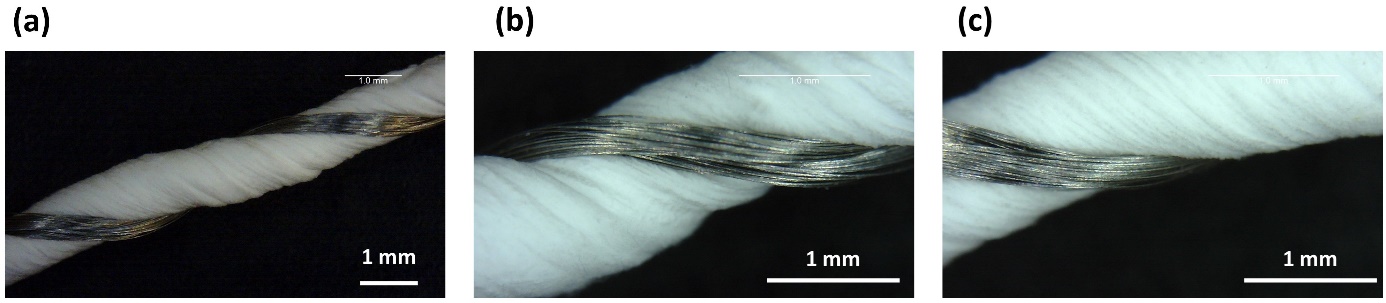


**Figure S26.** OM images of yarn device before PDMS coating.


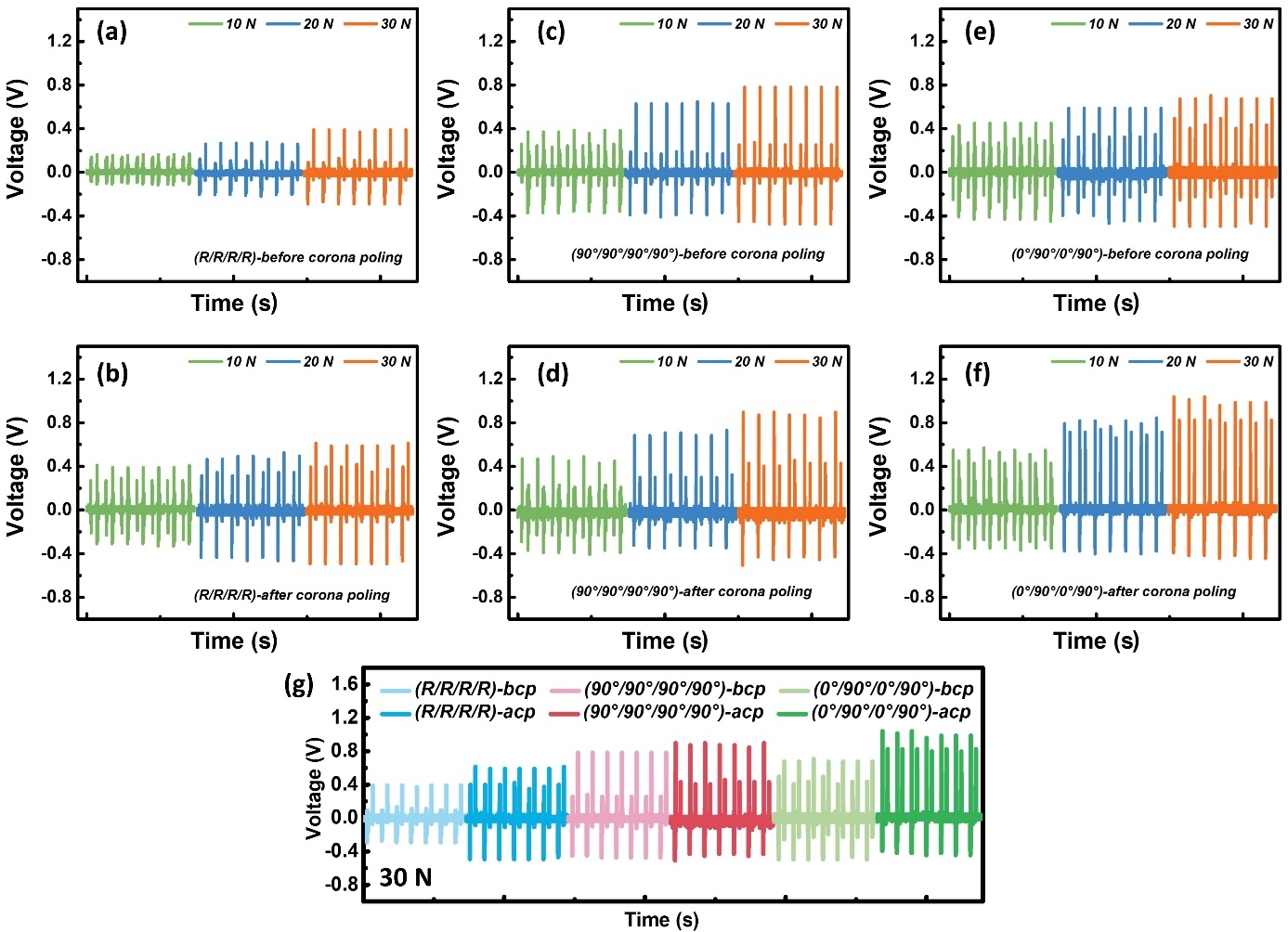


**Figure S27.** Effect of pushing force (10 N, 20 N, and 30 N) on the yarn device output voltage. (a) (R/R/R/R)-before corona poling. (b) (R/R/R/R)-after corona poling. (c) (90°/90°/90°/90°)-before corona poling. (d) (90°/90°/90°/90°)-after corona poling. (e) (0°/90°/0°/90°)-before corona poling. (f) (0°/90°/0°/90°)-after corona poling. (g) Pushing test results of (R/R/R/R), (90°/90°/90°/90°), (0°/90°/0°/90°) fiber yarns before and after corona poling (denoted as bcp and acp, respectively) under 30 N.


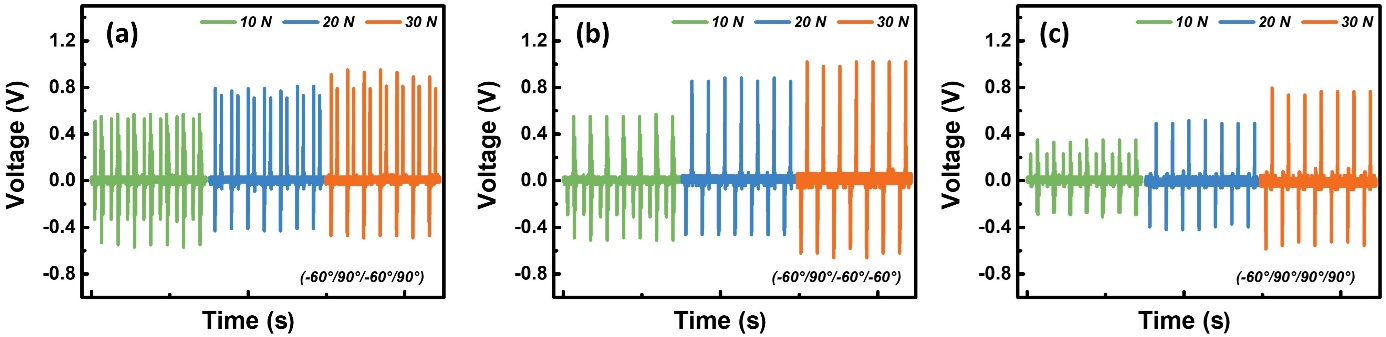


**Figure S28.** Effect of pushing force (10 N, 20 N, and 30 N) on the yarn device output voltage.

(a) (-60°/90°/-60°/90°)- after corona poling. (b) (-60°/90°/-60°/-60°)- after corona poling. (c) (-60°/90°/90°/90°)-after corona poling.

Figure S29 provides XRD characterization of the optimized yarns (-60°/90°/-60°/90°), (-60°/90°/-60°/-60°), (-60°/90°/90°/90°) after corona poling. The results similarly show that, despite different equilibrium points after optimization, the piezoelectric performance of the three sets of samples is maintained at similar levels.


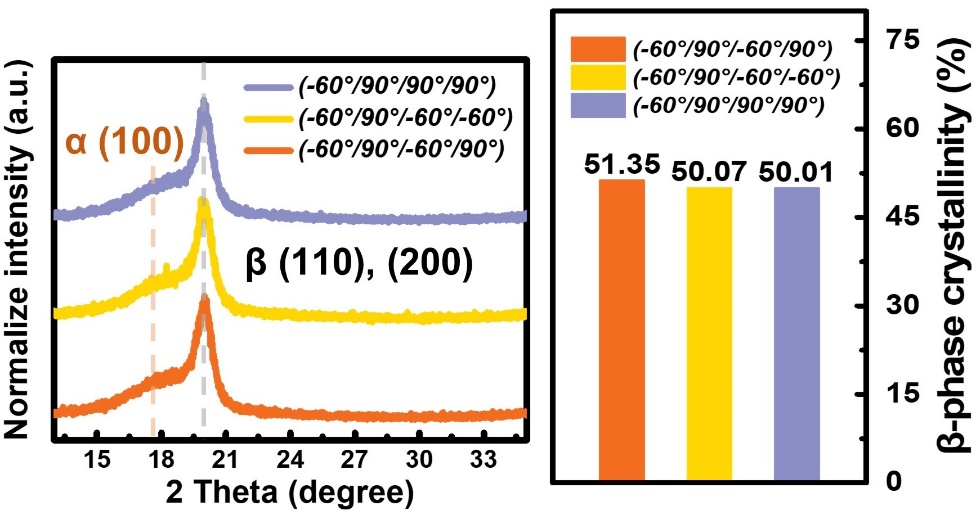


**Figure S29.** Optimized yarn (-60°/90°/-60°/90°), (-60°/90°/-60°/-60°), and (-60°/90°/90°/90°) after corona poling XRD pattern and *β*-phase crystallinity calculated by XRD measurement.

**6. Demonstration of Self-powered Yarn Sensing Applications**

The pre-curing time of the PDMS solution before coating is crucial, significantly affecting the morphology of the yarn device. If the recently prepared PDMS solution is used for coating, the yarn becomes slightly transparent (Figure S30b). We also conducted a pushing test on this transparent yarn device, and the results show that the transparent yarn device has no output (Figure S31). This is because, without pre-curing for a while, the strong fluidity of PDMS flows into the gap of the fiber, causing the yarn to become transparent. Simultaneously, because the fiber gap is filled with non-conductive PDMS, this significantly reduces the output of the yarn device or even leads to no output. Through comparison, we find that after pre-curing for 12 hours, the viscosity of PDMS increases, making it difficult to flow into the fiber gap before completely drying. The fibers retain their white appearance (Figure S30h).


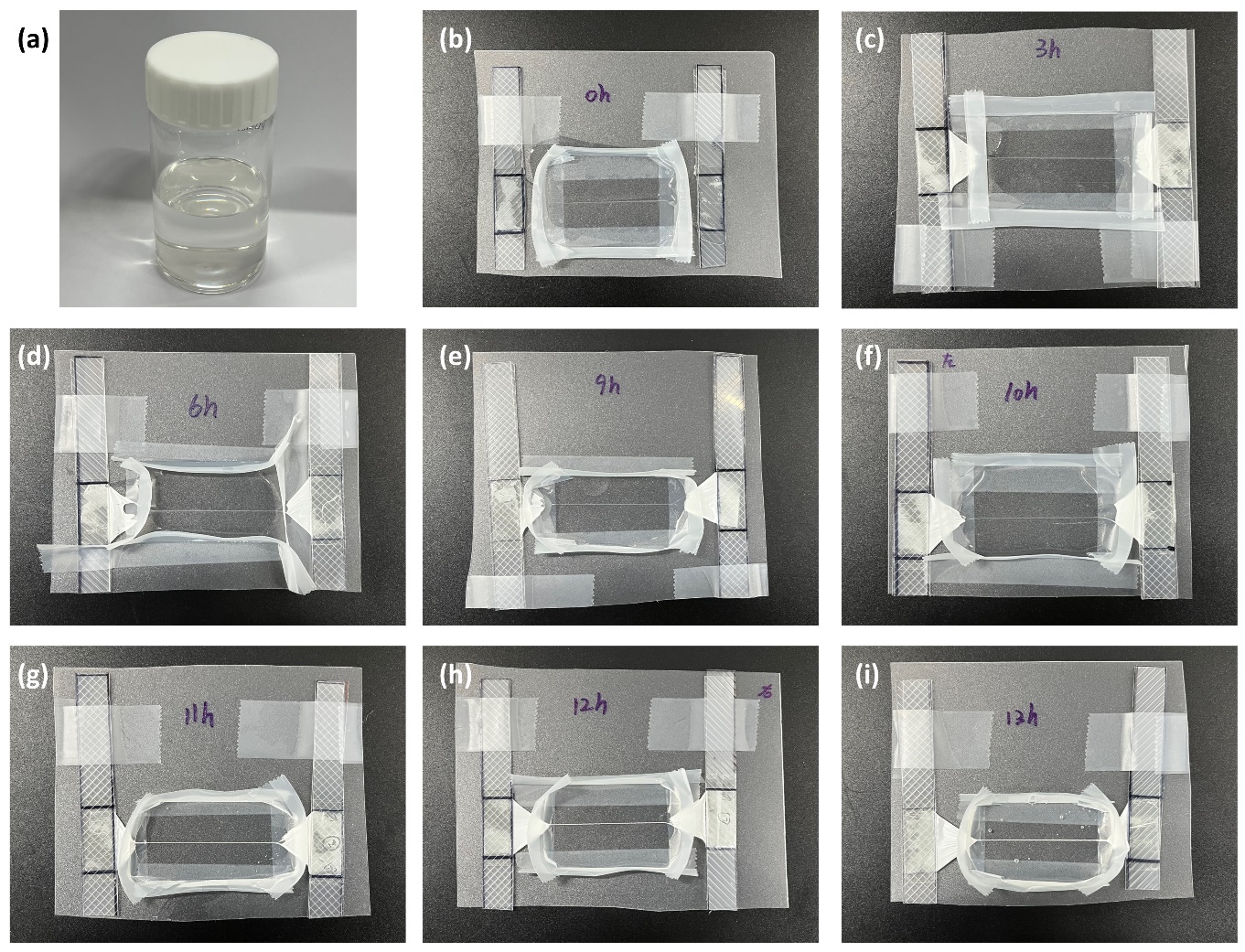


**Figure S30.** The effect on the transparency of the yarn after coating the yarn with PDMS has been kept at room temperature for some time after the production is completed. (a) Prepared PDMS solution. (b) 0 h. (c) 3 h. (d) 6 h. (e) 9 h. (f) 10 h. (g) 11 h. (h) 12 h. (i) 13 h.


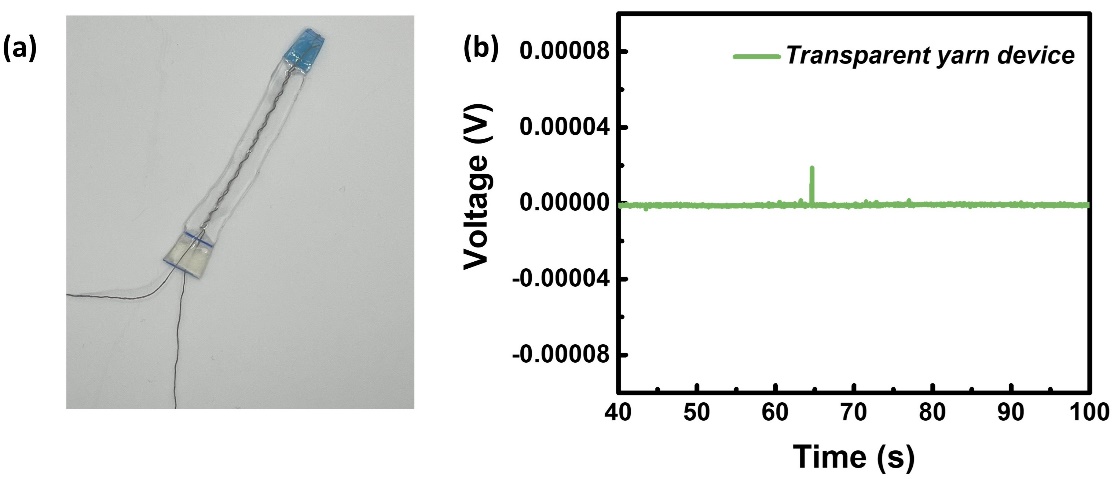


**Figure S31.** (a) Digital photo of transparent yarn device. (b) Pushing test output of transparent yarn device.


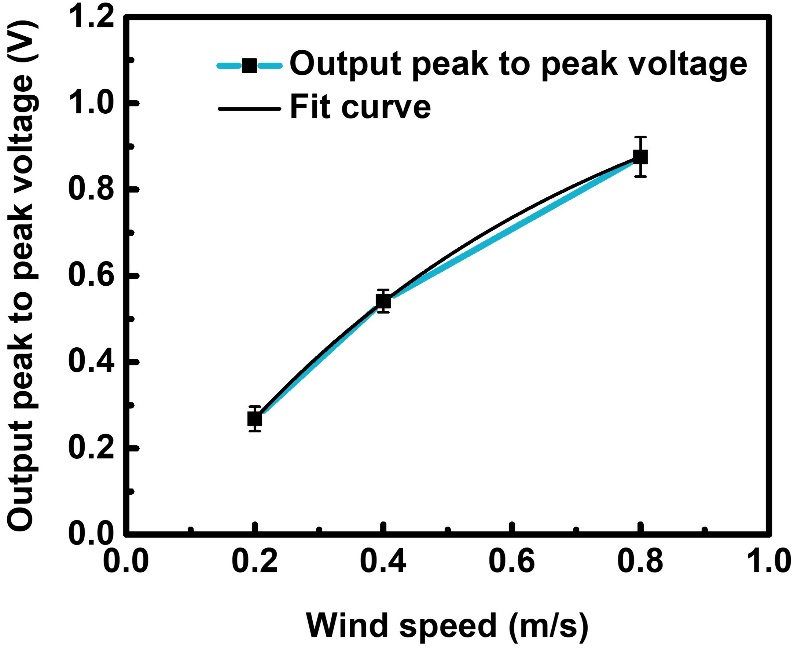


**Figure S32.** Wind speed and output voltage relationship curve and fitting curve.


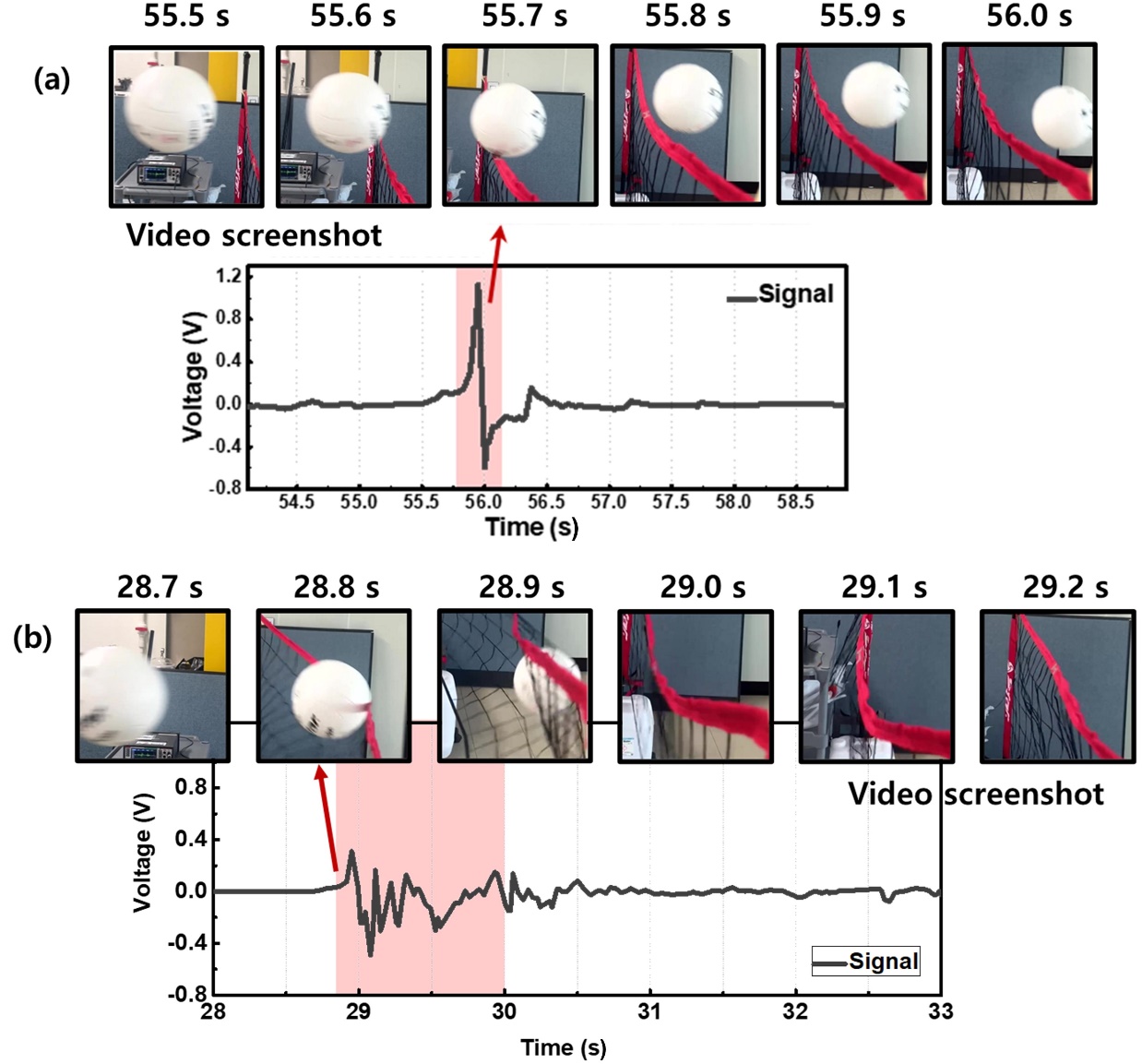


**Figure S33.** The output voltage of the yarn device for volleyball net touch detection.

**7. Experimental Details**

The final tensile test specimens for fiber mat samples are illustrated in Figure S34. Initially, the fiber mat is transferred onto paper and cut into rectangular pieces measuring 7 cm (length) by 1 cm (width). To ensure a secure grip on both ends of the fiber mat during the tensile experiment and prevent it from slipping out of the fixtures, a 2 cm section at each end of the fiber mat is doubly affixed to the paper frame using craft glue and double-sided tape, and the corresponding portion of the paper frame is then re-covered with an additional layer of paper. Subsequently, the specimens are left to dry at room temperature for over 24 h. As a result, the actual test section of the sample measures 3 cm (length) by 1 cm (width).

The sample preparation method for yarn samples is similar to that of fiber mats, with the main difference being that once the yarn is twisted, it is fixed directly in the middle of the paper frame and secured using the same method. The actual test dimensions for the yarn samples are 3 cm (length) by 0-1.5 mm (diameter).


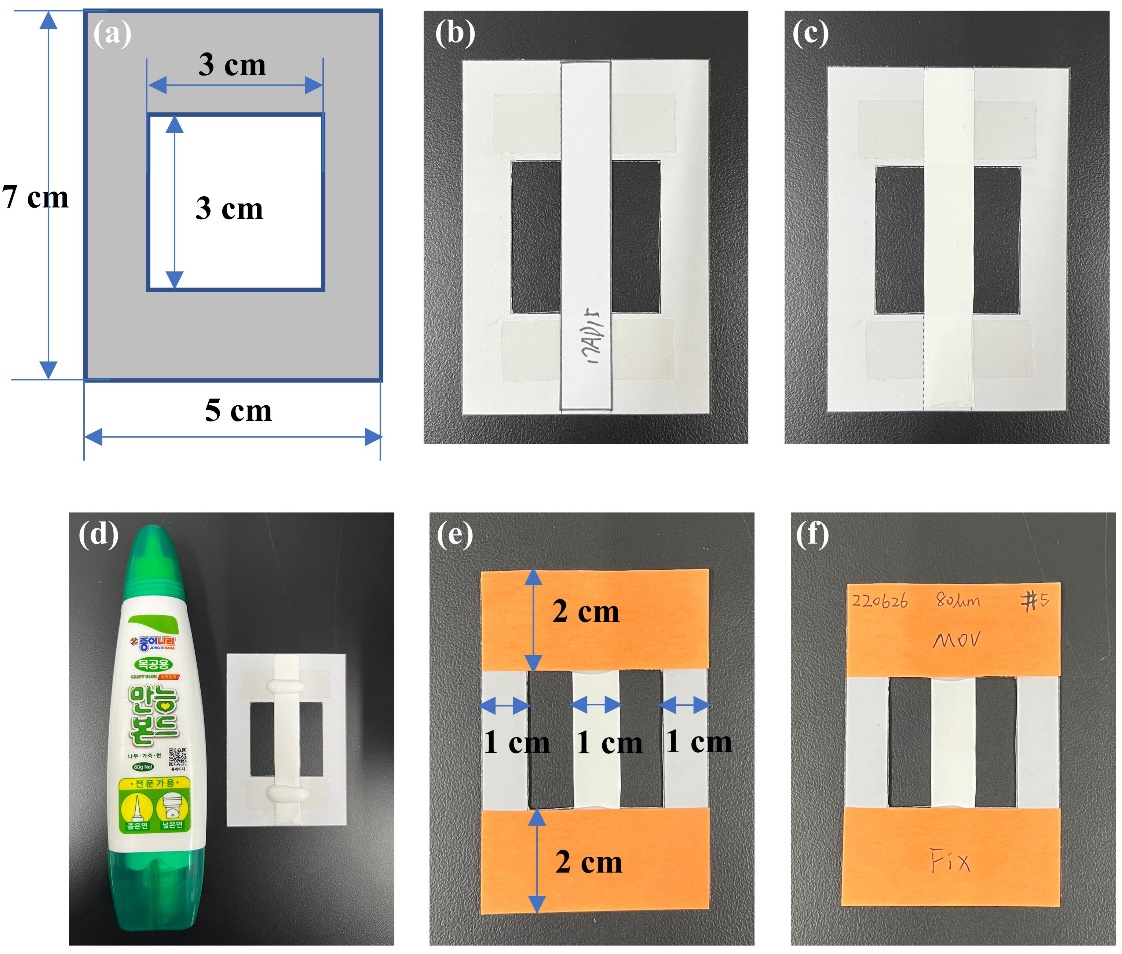


**Figure S34.** Mat tensile test specimen preparation process (a) Cut the A4 paper into a 5 cm × 7 cm square and cut a 3 cm × 3 cm area in the middle. (b), (c) Use double-sided tape to fix the fiber mat on the paper frame and remove the support paper. (d) Reinforce with a universal bond. (e), (f) Cover the universal bond with another paper, press the bond evenly, and make a mark. Finally, cut out the paper wall (left and right parts) before starting the tensile test.

**8. Resources used for the development of MBO Python script**

For the development of a Python script that performs the Multi-objective Bayesian optimization, we used relevant open-source libraries and made appropriate modifications to the libraries based on the algorithms described in the literature. Below we provide references to the open-source library and journal papers that we referred to:

**Open source Gaussian process regression library:**

- https://gpy.readthedocs.io/en/deploy/

**Non-continuous (Discrete) Gaussian process regression model algorithm:**

- E. C. Garrido-Merchán, D. Hernández-Lobato, Neurocomputing 2020, 380, 20.

**Application of Heteroscedastic GPR model:**

- https://nbviewer.org/github/SheffieldML/notebook/blob/master/GPy/heteroscedastic_regression.ipynb

**Implementation of Multi-objective acquisition function (EHVI):**

- K. Yang, M. Emmerich, A. Deutz and T. Ba¨ck, Efficient computation of expected hypervolume improvement using box decomposition algorithms, J. Glob. Optim., 2019, 75, 3–34.

**References**

[1] M. Kim, S. Lee, Y.-i. Kim, *APL Mater.* **2020**, 8.
